# Folate receptor α increases chemotherapy resistance through stabilizing MDM2 in cooperation with PHB2 that is overcome by MORAb‐202 in gastric cancer

**DOI:** 10.1002/ctm2.454

**Published:** 2021-06-01

**Authors:** Hitomi Sakai, Hisato Kawakami, Takeshi Teramura, Yuta Onodera, Elizabeth Somers, Keiji Furuuchi, Toshimitsu Uenaka, Ryoji Kato, Kazuhiko Nakagawa

**Affiliations:** ^1^ Department of Medical Oncology Kindai University Faculty of Medicine Osaka‐Sayama Osaka Japan; ^2^ Division of Cell Biology for Regenerative Medicine Institute of Advanced Clinical Medicine Kindai University Faculty of Medicine Osaka‐Sayama Osaka Japan; ^3^ AD Franchise Special Mission, Eisai Inc. Woodcliff Lake New Jersey USA; ^4^ Epochal Precision Anti‐Cancer Therapeutics (EPAT), Eisai Inc. Exton Pennsylvania USA

**Keywords:** antibody‐drug conjugate, folate receptor α (FOLRα), gastric cancer, MDM2

## Abstract

**Background:**

The main function of folate receptor α (FOLRα) has been considered to mediate intracellular folate uptake and induce tumor cell proliferation. Given the broad spectrum of expression among malignant tumors, including gastric cancer (GC) but not in normal tissue, FOLRα represents an attractive target for tumor‐selective drug delivery. However, the efficacy of anti‐FOLRα monoclonal antibodies (mAbs) has not been proved so far, with the reason for this failure remaining unclear, raising the need for a better understanding of FOLRα function.

**Methods:**

The distribution of FOLRα in GC cells was evaluated by immunohistochemistry. The impacts of FOLRα expression on the survival of GC patients and GC cell lines were examined with the Gene Expression Omnibus database and by siRNA of FOLRα. RNA‐sequencing and Microarray analysis was conducted to identify the function of FOLRα. Proteins that interact with FOLRα were identified with shotgun LC‐MS/MS. The antitumor efficacy of the anti‐FOLRα mAb farletuzumab as well as the antibody‐drug conjugate (ADC) consists of the farletuzumab and the tublin‐depolymerizing agent eribulin (MORAb‐202) was evaluated both *in vitro* and *in vivo*.

**Results:**

FOLRα was detected both at the cell membrane and in the cytoplasm. Shorter overall survival was associated with FOLRα expression in GC patients, whereas reduction of FOLRα attenuated cell proliferation without inducing cell death in GC cell lines. Transcriptomic and proteomic examinations revealed that the FOLRα‐expressing cancer cells possess a mechanism of chemotherapy resistance supported by MDM2, and FOLRα indirectly regulates it through a chaperone protein prohibitin2 (PHB2). Although reduction of FOLRα brought about vulnerability for oxaliplatin by diminishing MDM2 expression, farletuzumab did not suppress the MDM2‐mediated chemoresistance and cell proliferation in GC cells. On the other hand, MORAb‐202 showed significant antitumor efficacy.

**Conclusions:**

The ADC could be a more reasonable choice than mAb as a targeting agent for the FOLRα‐expressing tumor.

## Graphical abstract

1. MDM2 may determine the malignant phenotype of FOLRα‐expressing cancers as a result of the FOLRα‐PHB2‐MDM2 axis.
2. The anti‐FOLRα monoclonal antibody farletuzumab did not suppress the MDM2‐mediated chemotherapy resistance and cell proliferation in gastric cancer cells.
3. The antitumor efficacy of MORAb‐202, a farletuzumab‐eribulin antibody‐drug conjugate, for gastric cancer cell lines depended on the level of FOLRα expression.

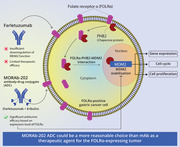

**Abbreviations**
ADC: antibody‐drug conjugate
EMT: epithelial mesenchymal transition
ERK: extracellular signal‐regulated Kinase
FACS: fluorescence‐activated cell sorting
FBS: fetal bovine serum
FCS: Fetal Calf Serum
FITC: fluorescein isothiocyanate
FOLRα: folate receptor α
GC: gastric cancer
GEO: gene expression omnibus
GO: gene ontology
GPI: glycosylphosphatidylinositol
HRP: horseradish peroxidase
LC‐MS/MS: liquid chromatography tandem mass spectrometry
mAb: monoclonal antibody
MAPK: mitogen‐activated protein kinase
PHB1: prohibitin1
PHB2: prohibitin2
RT‐qPCR: reverse transcription‐quantitative polymerase chain reaction

## 1 INTRODUCTION

Gastric cancer is the fifth most common cancer type and the third leading cause of cancer deaths worldwide.^1^ Given the limited number of selective and effective molecularly targeted agents available for gastric cancer, the identification of new target molecules is urgently needed.

Folate receptor α (FOLRα) is a 38‐kDa glycosylphosphatidylinositol (GPI)–linked cell‐surface glycoprotein encoded by the gene *FOLR1*. FOLRα binds folate with high affinity and mediates its cellular uptake.^2^ FOLRα is expressed in various tumor types, including ovarian, endometrial, non–small cell lung, and triple‐negative breast cancers, whereas its expression at a substantial level in normal tissues is essentially limited to the kidney, choroid plexus, placenta, and lung.^3^ This expression pattern—high expression in a broad spectrum of solid tumors and low expression in normal tissue—has led to an interest in FOLRα as a potential therapeutic target. Indeed, FOLRα‐targeting agents have been preceded by treatment of patients with the four malignancies with the highest rates of FOLRα overexpression: ovarian, endometrial, triple‐negative breast, and non‐small cell lung cancer.^4^ Gastric cancer can be another candidate of anti‐FOLRα therapy given a substantial rate of FOLRα expression.^5^

Among FOLRα‐targeting agents, farletuzumab, a humanized monoclonal antibody (mAb), is the most advanced in clinical development. In a phase III study of platinum‐sensitive ovarian cancer, however, the addition of farletuzumab to carboplatin plus either paclitaxel or docetaxel failed to improve treatment outcome, with the reason for this failure remaining unclear.^4^ Nonetheless, the concept of FOLRα‐targeted therapy continues to be explored with multiple anti‐FOLRα agents—including MORAb‐202, an antibody‐drug conjugate (ADC) consisting of farletuzumab and the tubulin inhibitor eribulin mesylate—with some of these agents have entered clinical trials.^4^ A better understanding of FOLRα function would facilitate the development of such therapy.

We have now studied the role of FOLRα in gastric cancer and found that a signaling axis comprising FOLRα, prohibitin 2 (PHB2), and murine double minute 2 (MDM2) contributes to chemotherapy resistance. We further found that this resistance mechanism could be overcome by MORAb‐202, although not by farletuzumab plus chemotherapy. Together with the substantial frequency of FOLRα expression in gastric cancer, our results suggest that FOLRα is indeed a feasible therapeutic target for gastric tumors.

## 2 METHODS

### 2.1 Cells and reagents

The human gastric cancer cell lines MKN1 (RRID, CVCL_1415), MKN74 (CVCL_2791), MKN45 (CVCL_0434), and NUGC3 (CVCL_1612), as well as HeLa cervical carcinoma cells (CVCL_0030), were obtained from the JCRB cell bank. The human gastric cancer cell lines SNU1 (CVCL_0099), Hs746T (CVCL_0333), and NCI‐N87 (CVCL_1603) were from American Type Culture Collection and SNU216 (CVCL_3946) was from the Korean Cell Line Bank. The cells were maintained under a humidified atmosphere of 5% CO_2_ at 37°C in RPMI 1640 medium (Sigma‐Aldrich) supplemented with 10% heat‐inactivated fetal bovine serum (FBS) (Biowest) and 1% penicillin‐streptomycin‐amphotericin B (Wako). Cells were tested for mycoplasma contamination using MycoAlert (LT07, Lonza) and were confirmed negative. Eribulin was obtained from Eisai Co. Ltd and Oxaliplatin from Yakult. MORAb‐202 and farletuzumab were provided by Eisai Co. Ltd. MG132 was obtained from Funakoshi.

### 2.2 Antibodies and primers

Sequences of PCR primers, guide RNAs, and siRNAs used in this study are shown in Table 1. Antibodies and dilution conditions are shown in Table 2.

**TABLE 1:** Sequences of PCR primers, guide RNAs, and siRNAs used in this study

RT‐qPCR primers	Forward (5′‐3′)	Reverse (5′‐3′)
*FOLR1*	TTCATCCAGGACACCTGCCTC	ATTGCTCACAGTCCTCTTTGC
*MDM2*	AACAGGTGTCACCTTGAAGGTG	TGAGGTAGATGGTCTAGAAACC
*GAPDH*	TGGTAAAGTGGATATTGTTGC	TTCTCAGCCTTGACGGTGC
**Genotyping PCR primers**		
*FOLR1*	TCAGGTGATCCACCCACCTC	AGATCTTTGGAGGAGTCATTC
**Guide RNAs**	**Sense (5′‐3′)**	**Antisense (5′‐3′)**
FOLR1‐1	ACACC ACCTGAACCTCGTGACCACC G	AAAAC GTGGTCACGAGGTTCAGGTC G
FOLR1‐2	ACACC GTTGGCATTGTACCGACATT G	AAAAC AATGTCGGTACAATGCCAAC G
**siRNAs**		
*FOLR1*	GGA UGU UUC CUA CCU AUA UdTdT	AUA UAG GUA GGA AAC AUC CdTdT
*MDM2*	GAA AAU UCA GAU GAA UUA UdTdT	AUA AUU CAU CUG AAU UUU CdTdT
*PHB1*	GCA AAG AUU UAC AGA AUG UdTdT	ACA UUC UGU AAA UCU UUG CdTdA
*PHB2*	CAG AAU CGU AUC UAU CUC AdTdT	UGA GAU AGA UAC GAU UCU GdTdT
Scrambled	GUA CUC AUG CUA UAU UGC UdTdT	AGC AAU AUA GCA UGA GUA CdTdT

**TABLE 2:** Antibodies and dilution conditions

Antibodies	Company	Application	Dilution
PHB1 (#2426)	Cell Signaling Technology	IB	1/1000 in Immunoenhancer
PHB2 (E1Z5A, #14085)	Cell Signaling Technology	IB	1/1000 in Immunoenhancer
p53 (#9282)	Cell Signaling Technology	IB	1/5000 in TBS containing 0.2% Tween‐20 and 10% Block Ace
MDM2 (D1V2Z, #86934)	Cell Signaling Technology	IB	1/1000 in Immunoenhancer
Rb (D20, 9313)	Cell Signaling Technology	IB	1/1000 in Immunoenhancer
Phospho–histone H3 (Ser^10^) (#3377, D2C8)	Cell Signaling Technology	IB	1/1000 in Immunoenhancer
Phospho‐Wee1 (Ser^642^) (#4910, D47G5)	Cell Signaling Technology	IB	1/1000 in Immunoenhancer
CDK2 (#2546, 78B2)	Cell Signaling Technology	IB	1/1000 in Immunoenhancer
CDK4 (#12790, D9G3E)	Cell Signaling Technology	IB	1/1000 in Immunoenhancer
CDK6 (#3136, DCS83)	Cell Signaling Technology	IB	1/1000 in Immunoenhancer
Myt1 (#4282)	Cell Signaling Technology	IB	1/1000 in Immunoenhancer
p27(Kip1) (#3686, D69C12)	Cell Signaling Technology	IB	1/1000 in Immunoenhancer
Cyclin D3 (#2936, DCS22)	Cell Signaling Technology	IB	1/1000 in Immunoenhancer
p21(Waf1/Cip1) (#2947, 12D1)	Cell Signaling Technology	IB	1/1000 in Immunoenhancer
Cyclin A2 (#4656, BF683)	Cell Signaling Technology	IB	1/1000 in Immunoenhancer
Cyclin B1 (#12231, D5C10)	Cell Signaling Technology	IB	1/1000 in Immunoenhancer
Phospho‐CDC2 (Tyr^15^) (#4539, 10A11)	Cell Signaling Technology	IB	1/1000 in Immunoenhancer
Cyclin E2 (#4132)	Cell Signaling Technology	IB	1/1000 in Immunoenhancer
p18(Ink4c) (#2896, DCS118)	Cell Signaling Technology	IB	1/1000 in Immunoenhancer
Cyclin D1(#2978, 92G2)	Cell Signaling Technology	IB	1/1000 in Immunoenhancer
Phospho‐GSK3β (Ser^9^) (#9323, 5B3)	Cell Signaling Technology	IB	1/1000 in Immunoenhancer
EP300 (#86377, D8Z4E)	Cell Signaling Technology	IB	1/1000 in Immunoenhancer
FOLRα (NCL‐L‐FRα )	Leica	IB	1/1000 in Immunoenhancer
Phospho‐ATM (Ser^1981^) (2152‐1)	Epitomics	IB	1/1000 in Immunoenhancer
GAPDH (016‐25523, 5A12)	FujiIFilm Wako	IB	1/1000 in Immunoenhancer
HRP‐conjugated goat anti–mouse IgG (G0407)	Tokyo Chemical Industry	IB	1/1000 in Immunoenhancer
HRP‐conjugated goat anti–rabbit IgG (G0418)	Tokyo Chemical Industry	IB	1/1000 in Immunoenhancer
Phospho p38 (Thr180/Tyr182, #4511)	Cell Signaling Technology	IB	1/3000 in Immuno‐enhancer
p38 (#9212)	Cell Signaling Technology	IB	1/5000 in Immuno‐enhancer
Phospho Erk1/2	Cell Signaling Technology	IB	1/3000 in Immuno‐enhancer
Erk1/2 (#4695)	Cell Signaling Technology	IB	1/5000 in Immuno‐enhancer
Phospho Akt (Ser473, #4060)	Cell Signaling Technology	IB	1/3000 in Immuno‐enhancer
Akt (#9272)	Cell Signaling Technology	IB	1/5000 in Immuno‐enhancer
Caspase‐3 (#9662)	Cell Signaling Technology	IB	1/3000 in Immuno‐enhancer
FOLRα (NCL‐L‐FRα)	Leica	IP for IB	3 μg
MDM2 (sc‐813, N‐20)	Santa Cruz Biotechnology	IP for IB	3 μg
Normal human IgG (143‐09501)	FujiFilm Wako	IP for IB	3 μg
Normal rabbit IgG (PM035)	MBL	IP for IB	3 μg
FOLRα (NCL‐L‐FRα)	Leica	IP for MS	1 μg
Normal human IgG (143‐09501)	FujiFilm Wako	IP for MS	1 μg
FOLRα (NCL‐L‐FRα)	Leica	Flow cytometry /FACS	10 μg/mL

Abbreviations. IB, immunoblot analysis; IP, immunoprecipitation; MS, mass spectrometry; FACS, fluorescence‐activated cell sorting.

### 2.3 Animal use and care

All animal experiments were performed following the Recommendations for Handling of Laboratory Animals for Biomedical Research compiled by the Committee on Safety and Ethical Handling Regulations for Laboratory Animal Experiments, Kindai University. The study was also approved by the Animal Ethics Committee of Kindai University. Female BALB/cAJcl‐nu/nu mice (CLEA Japan) were housed in groups of four or five with food and water available *ad libitum*, and they were exposed to an artificial light‐dark regimen with 14 h of light and 10 h of darkness and to a temperature maintained between 20°C and 25°C in a ventilated room.

### 2.4 Immunohistochemistry method for staining and interpretation of results in the formalin‐fixed, paraffin‐embedded whole tissue sections to evaluate the frequency of expression in the Tumor Scan Study

FOLRα expression levels were determined using the FRA‐26B3 IHC assay kit manufactured by Biocare Medical, Inc. and stained using the Biocare intelliPath Automated Slide Stainer. The following assay procedure was utilized for staining endometrial, gastric, triple‐negative breast cancer, non‐small cell lung adenocarcinoma, and ovarian carcinoma formalin‐fixed, paraffin‐embedded tissue sections. Following incubation with the primary monoclonal antibody to human FOLRα protein or the negative control reagent, a rabbit anti‐mouse secondary polyclonal antibody was used to detect the primary antibody, and horseradish peroxidase (HRP)‐labeled goat polyclonal anti‐rabbit micro‐polymer was used to recognize the rabbit immunoglobulin present in the secondary antibody. The subsequently added 3,3′‐diaminobenzidine chromogen is converted by the HRP enzyme of the micro‐polymer into a visible reaction product (brown precipitate) at the antigen site. The specimens were counterstained, dehydrated, cleared, and cover‐slipped for scoring and interpretation by a board‐certified anatomic pathologist using a light microscope.

Interpretation of FOLRα expression levels was accomplished using specific criteria for evaluation. Each slide was evaluated for both the percentage of membrane and cytoplasm stained and the intensity of staining. Results of cytoplasmic and membranous expression levels were both recorded. Negative expression levels were scored as 0, for no staining present; 1+, for weak staining; 2+, for moderate and 3+, for strong staining. Only tumor cells were scored. FOLRα positivity was defined as membrane staining greater than or equal to 5% of neoplastic cells at any intensity level. Cytoplasmic staining levels were evaluated and recorded for use in the evaluation and characterization of staining; however, they were not utilized in the determination of percent positive results included in the tumor expression figure.

### 2.5 Propidium iodide staining

Cells were transfected with *si*FOLRα or treated with oxaliplatin (L‐OHP) for 48 h. Cells were incubated with propidium iodide (PI) at a concentration of 0.1 mg/ml in 0.01 M phosphate‐buffered saline (PBS), pH 7.2, for 30 min at room temperature in the dark. Cells were thoroughly washed in PBS, mounted in glycerol, and observed under a fluorescence microscope.

### 2.6 Immunofluorescence analysis

Cells were fixed for 30 min at room temperature with 10 N Mildform (Wako), washed three times with TBS containing 0.1% Tween‐20, incubated for 30 min in Block Ace, and washed again twice before exposure overnight at 4°C to antibodies to FOLRα. The cells were washed another three times, incubated for 60 min at room temperature with fluorescein isothiocyanate (FITC)–conjugated secondary antibodies, and then stained with DAPI before observation with a BZX‐710 microscope (Keyence).

### 2.7 Cell counts

Cells were plated at a density of 5 × 10^4^ per well of a 24‐well plate and incubated for the indicated times with the indicated agents, that is, oxaliplatin or eribulin for 24 h. The cells were then isolated as single‐cell suspensions by exposure to trypsin, and they were counted with a hemocytometer after staining with trypan blue (Thermo Fisher Scientific).

### 2.8 In vitro cell viability assay

Cells were plated in 96‐well flat‐bottomed plates at a density of 1200 to 6000 per well depending on the cell line in RPMI 1640 medium supplemented with 10% FBS. After culturing for 24 h, the cells were exposed to various concentrations of MORAb‐202 for 120 h, and cell viability was then assessed with the use of a Cell Counting Kit‐8 (Promega).

### 2.9 Reverse transcription‐quantitative polymerase chain reaction analysis

Total RNA was extracted from cells using the TRIzol reagent (Molecular Research Center) and was subjected to RT with a PrimeScript RT Master Mix Kit (Takara Bio). The resulting cDNA was subjected to real‐time PCR analysis with the use of a Thermal Cycler Dice Real Time System Single, with incubation at 95°C for 20 s followed by 40 cycles of 95°C for 5 s and 60°C for 30 s. For quantitation of relative gene expression, the threshold cycle (Ct) values were normalized by that for the housekeeping gene GAPDH and were then calibrated with the ΔΔCt method. Amplification of contaminating genomic DNA was limited with the use of primers designed to span at least one intron. Primer sequences are listed in Table 1.

### 2.10 Immunoblot analysis

Cells were homogenized in SDS sample buffer [4% SDS, 125 mM Tris‐glycine, 10% β‐mercaptoethanol, and 2% bromophenol blue in 30% glycerol], the homogenate was centrifuged at 15,000 rpm at 4°C for 10 min to remove debris, and portions of the resulting supernatant were subjected to SDS‐PAGE. The separated proteins were transferred to a polyvinylidene difluoride membrane (GE Healthcare), which was then exposed overnight at room temperature to Block Ace (Dainippon Sumitomo Pharma) before incubation overnight at 4°C with primary antibodies. Immune complexes were detected with horseradish peroxidase‐conjugated secondary antibodies and Immunostar LD (Wako) reagents. All antibodies used are listed in Table 2.

### 2.11 Co‐immunoprecipitation analysis

Cells were lysed in IP extraction buffer [25 mM Tris‐HCl (pH 7.5), 100 mM NaCl, 0.5% Triton X‐100] for 30 min on ice, the lysate was centrifuged to remove debris, and the resulting supernatant was incubated overnight at 4°C with primary antibodies conjugated to Dynabeads Protein G (Veritas) in Tris‐buffered saline (TBS) containing 0.02% Tween‐20. The precipitated proteins were eluted in an SDS sample buffer for immunoblot analysis. All antibodies used are listed in Supplementary Table 2.

### 2.12 Liquid chromatography–tandem mass spectrometry analysis

HeLa cells were fixed for 10 min at room temperature with 0.1% formaldehyde (Wako) and then lysed with a modified RIPA buffer consisting of 20 mM HEPES‐NaOH (pH 7.5), 1 mM EGTA, 1 mM MgCl_2_, 150 mM NaCl, 0.25% sodium deoxycholate, 0.05% SDS, 1% Nonidet P‐40, PhosSTOP (Merck, #4906845001), and Benzonase (1/500; Merck, #E1014). The lysate was subjected to immunoprecipitation as described above. The immunoprecipitate was collected in the modified RIPA buffer and was subjected to alkylation followed by digestion overnight at 37°C with Lys‐C (4 μg/ml) and Mass Spec Grade Trypsin (Promega). After desalting with GL‐Tip SDB (GL Sciences, #7820‐11200), the sample was concentrated with the use of a SpeedVac (Thermo Fisher Scientific), and its peptide concentration was determined with a Pierce Quantitative Colorimetric Peptide Assay Kit (Thermo Fisher Scientific, #23275). Peptides were fractionated with the use of a HyperSep Retain CX column and ammonium acetate. The fractions were then desalted with a C18 before analysis with Orbitrap Fusion Lumos and Q Exactive HF mass spectrometers. Spectral data files were analyzed with XlinkX 2.0 and Proteome Discover 2.2 software with the use of XlinkX node and SEQUEST HT. Binding proteins with a high confidence level (206 proteins) were selected from the raw data on the basis of the false discovery rate. Ribosomal proteins were then excluded, and the function of the remaining proteins (188 molecules) was examined by WikiPathway analysis. Sequential selection of proteins whose function is related to cell proliferation or cell survival and of proteins that are localized to the cell membrane or cytoplasm yielded 53 and 20 proteins.

### 2.13 RNA interference

Transfection of cells with siRNAs was performed with the use of the Lipofectamine RNAiMAX reagent (Thermo Fisher Scientific). The sequences of the siRNAs are provided in Table 1. Knockdown efficiency was confirmed by reverse transcription‐quantitative polymerase chain reaction (RT‐qPCR) or immunoblot analysis.

### 2.14 CRISPR/CAS9

For the CRISPR‐Cas9 treatment, MKN1 was dissociated with TrypLE Express, washed twice with Opti‐MEM, and resuspended in 100 μL of Opti‐MEM containing 7.5 μg of MLM3636 guide RNA expression plasmids and 5 μg Cas9 expression plasmid. MLM3636 plasmid was a gift from Dr. Keith Joung (Addgene plasmid # 43860) and pSpCas9(BB)‐2A‐Puro (PX459) V2.0 was a gift from Dr. Feng Zhang (Addgene plasmid # 62988). After 1 μg/μL puromycin selection for 10 days, the resulting colonies were cloned into the 96 well plates and genotyped. For genomic PCR for genotyping, the cells were digested by KAPA Express Extract (Nippon Genetics Co, Ltd., Tokyo, Japan) diluted in Tris‐EDTA. Genomic PCR was performed using KOD FX Neo DNA polymerase (TOYOBO Co. Ltd., Osaka, Japan) with genotyping primers.

### 2.15 PiggyBac transposon vector system

FOLR1 stably expressing cell lines were produced by introducing the pPB‐CAG‐FOLR1‐IRES‐Puro vector into MKN1 also used above. The FOLR1 cDNA obtained by KOD Plus NEO DNA polymerase was cloned into the EcoRI‐digested site of the pPB‐CAG‐IRES‐Puro plasmid to create the pPB‐CAG‐FOLR1‐IRES‐Puro vector. The pPB‐CAG‐FOLR1‐IRES‐Puro vector was electroporated into 1 × 10^6^ MKN1 using a NEPA21 electroporation system (NEPAGENE, Chiba, Japan) with a plasmid cording the piggyBac transposase (PBase). The transfected cells were cultured in 10% FCS (Foetal Calf Serum)‐DMEM supplemented with 10 ng/ml puromycin for 10 days.

### 2.16 In vivo tumor growth assay

Gastric cancer cell lines (1 × 10^7^ cells per mouse) were injected subcutaneously into the right flank of 6‐week‐old female BALB/cAJcl‐nu/nu mice. After tumors had achieved a target volume of 100 to 200 mm^3^, mice were randomly assigned to treatment groups and received a single intraperitoneal injection of PBS (100 μl, control) or of MORAb‐202 (5 mg/kg) or an equivalent molar dose of eribulin (0.1 mg/kg) (day0). The dose of MORAb‐202 was based on the results of a previous in vivo study.^6^ The molecular masses of MORAb‐202, eribulin mesylate, and farletuzumab are 155.2 kDa, 826.0 Da, and 29.8 kDa, respectively. Calculations were performed with a drug to antibody ratio for MORAb‐202 of 4. Tumor volume was measured twice a week, and body weight was measured once or twice a week.

The antitumor effects of MORAb‐202 versus PBS were demonstrated with the tumors that were excised from mice on day11, and tumor sections were prepared and fixed in formalin for subsequent experiments.

### 2.17 Hematoxylin and eosin staining

Tumor sections (5 μm) were stained with hematoxylin for 10 min, differentiated with 1% hydrochloric acid, and stained with eosin solution for 30 s. The sections were mounted on glass slides for pathological observations.

### 2.18 Survival analysis

An online tool (Kaplan‐Meier plotter, https://kmplot.com/analysis) was used to assess the relation between FOLR1 mRNA abundance and overall survival for gastric cancer patients. The patients were divided into two groups based on the expression level of FOLR1, with the cutoff being set automatically. The hazard ratio, its 95% confidence interval, and log‐rank *P*‐value were determined.

### 2.19 Statistical analysis

One‐way ANOVA followed by Tukey's honestly significant difference (HSD) test was performed with the use of GraphPad Prism 5 software. A *P*‐value of < .05 was considered statistically significant.

### 2.20 Additional methods

Additional methods are provided in Supporting Information Methods online.

## 3 RESULTS

### 3.1 FOLRα expression in gastric and other cancer types

**FIGURE 1:**
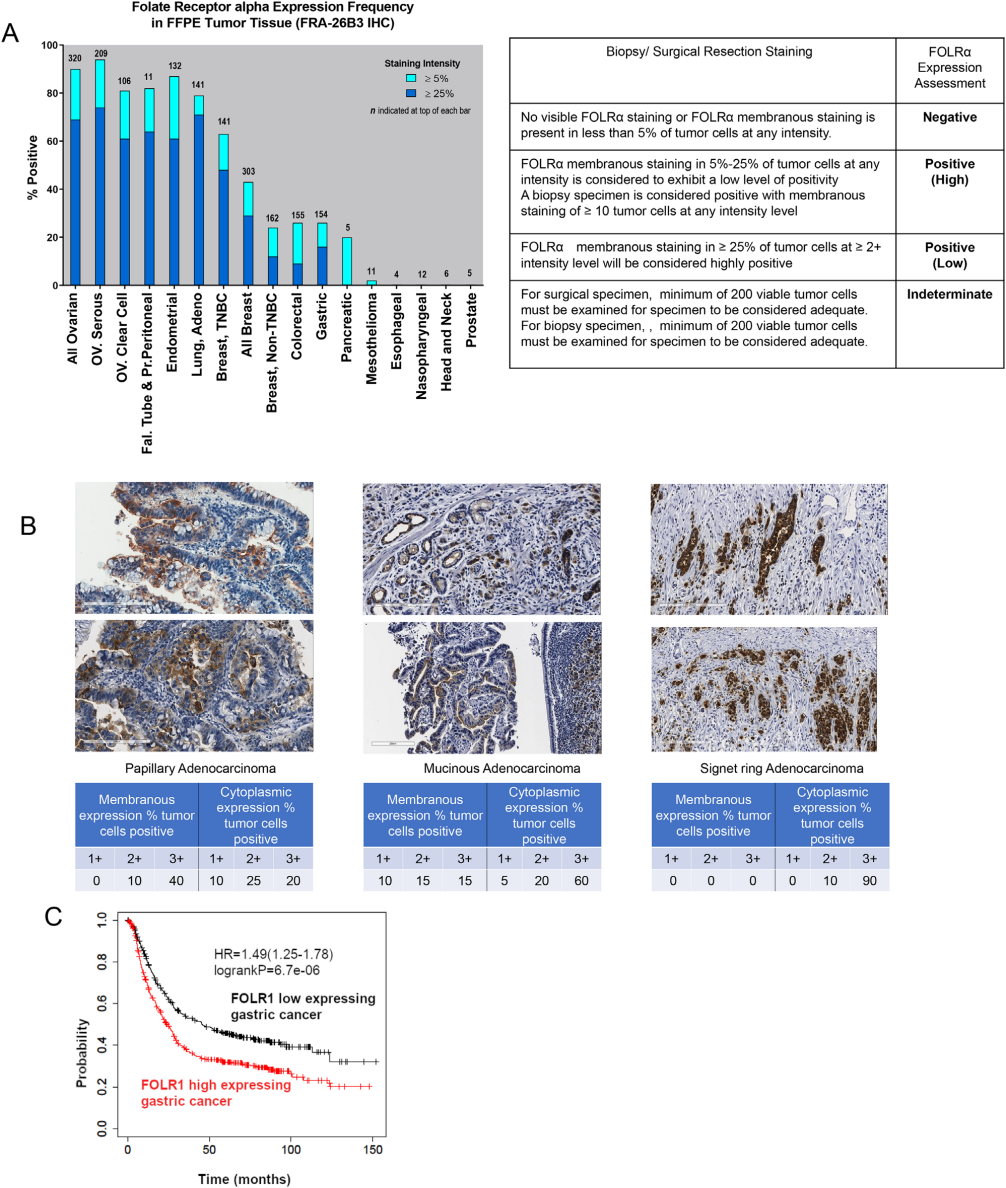
FOLRα expression in human gastric cancer. A, Frequency of FOLRα protein expression in various human cancer types. The percentage of tumors of each type positive for FOLRα expression was determined by immunohistochemistry. Dark and light blue shading correspond to tumors classified as strongly or weakly positive for FOLRα staining, as described in Supplementary Methods. The number at the top of each bar indicates the number of positive tumors. OV. Seous, ovarian serous carcinoma; OV. Clear Cell, ovarian clear cell carcinoma; Fal. tube & Pr.peritoneal, fallopian tube and primary peritoneal cancer; adeno, adenocarcinoma; TNBC, triple‐negative breast cancer. The table indicates scoring criteria for FOLRα immunohistochemical staining. B, Representative immunohistochemistry images of FOLRα staining in gastric cancer specimens. The percentages of tumor cells positive for membranous or cytoplasmic staining at each intensity level are shown below each image. Scale bars, 200 μm. C, Kaplan‐Meier curves of overall survival according to *FOLR1* expression level for 875 gastric cancer patients in the GEO database. The curves were generated with the use of Kaplan‐Meier plotter (http://kmplot.com/analysis). The hazard ratio (HR) with its 95% confidence interval as well as the log‐rank *P* value is shown

We first performed immunohistochemical analysis of FOLRα expression in various human cancer types, with gastric cancer having been shown to be among the tumor types with the highest rates of FOLRα positivity, after ovarian, endometrial, lung, and breast cancers.^4^ Among 133 gastric cancer specimens, 8.3% (11/133) were weakly positive, and 13.5% (18/133) were strongly positive for FOLRα expression, yielding a total positive rate of 21.8% (Figure 1A). FOLRα‐positive gastric tumors included all main histological subtypes (Table S1), and FOLRα staining was detected both at the cell membrane and in the cytoplasm (Figure 1B). We also examined the relationship between HER2 and FOLRα positivity in gastric cancer by tissue microarray analysis. The expression of FOLRα was more frequent among tumors positive for HER2 expression than among those negative [56.4% (22/39) versus 24.8% (25/101); *P* < .001, Pearson's Chi‐squared test].

Although a meta‐analysis previously showed that a high expression level of FOLRα is associated with poor survival in patients with ovarian, endometrial, lung, or breast cancer,^7^ the situation for patients with gastric cancer has been unclear. We, therefore, examined the possible impact of FOLRα expression on the survival of 875 gastric cancer patients in the Gene Expression Omnibus (GEO) database. Overall survival was significantly (*P* < .001) shorter for patients with a high level of *FOLR1* expression in their tumors than in those with a low level (Figure 1C), suggesting that FOLRα expression may be related to tumor aggressiveness in gastric cancer.

### 3.2 FOLRα promotes the proliferation of gastric cancer cells

**FIGURE 2:**
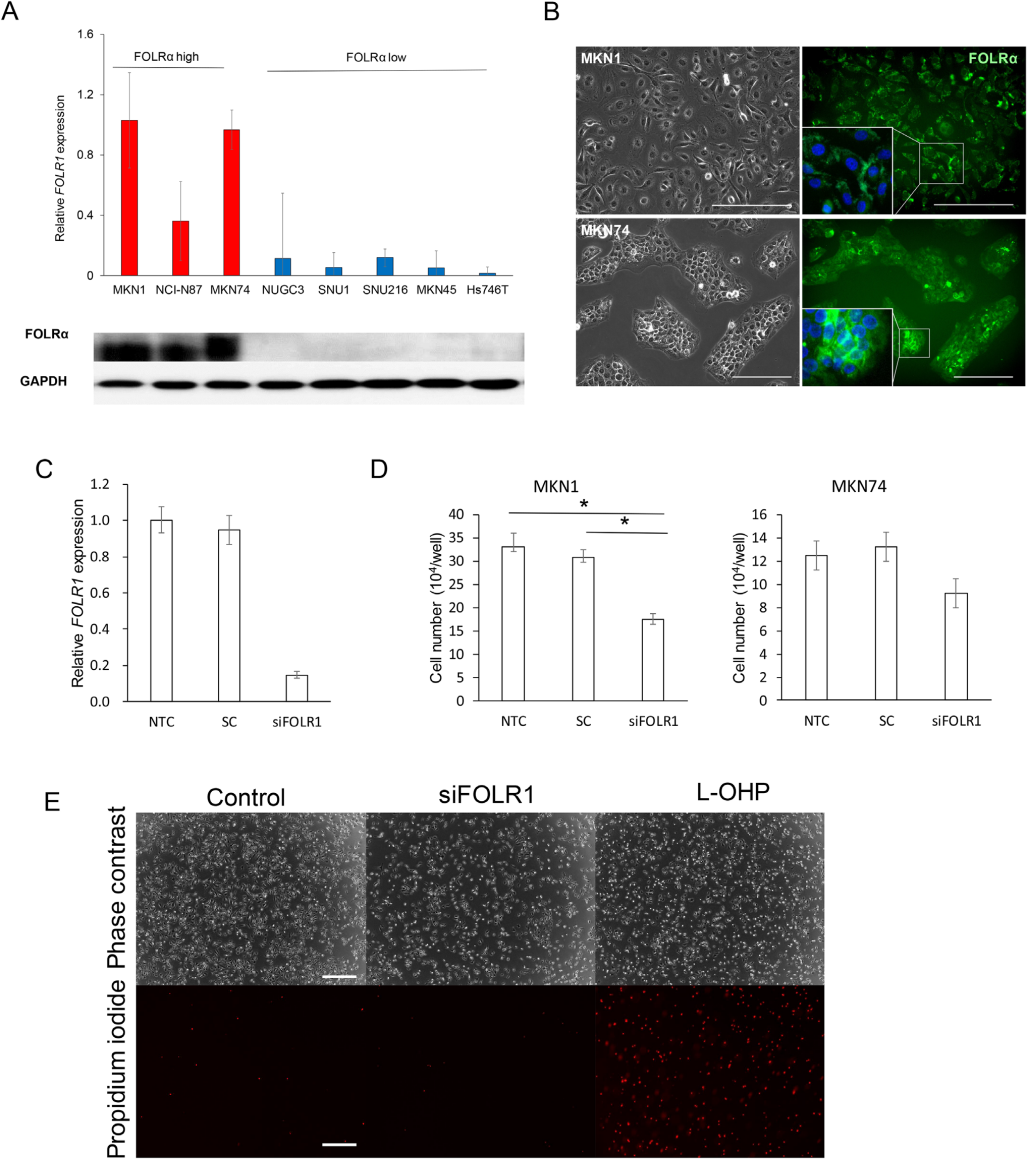
FOLRα expression and function in human gastric cancer cell lines. A, RT‐qPCR analysis of *FOLR1* mRNA and immunoblot analysis of FOLRα protein in eight gastric cancer cell lines. The RT‐qPCR data are means ± SD from four independent experiments. Glyceraldehyde‐3‐phosphate dehydrogenase (GAPDH) was examined as a loading control for immunoblot analysis. B, Immunofluorescence staining of FOLRα (green) in MKN1 and MKN74 cells (right). Nuclei were stained with 4′,6‐diamidino‐2‐phenylindole (DAPI, blue). Phase‐contrast images are also shown (left). Scale bars, 500 μm. C, RT‐qPCR analysis of *FOLR1* mRNA in MKN1 cells transfected (or not, NTC) with siFOLR1 or a scrambled control siRNA (SC). Data are means ± SD for four independent experiments. D, Cell number for MKN1 or MKN74 cells transfected as in C, plated at a density of 5 × 10^4^ cells per well, and then cultured for 24 h. Data are means ± SD for four independent experiments. *P < .05 (one‐way ANOVA followed by Tukey's post hoc test). E, Viability of MKN1 cells treated as in D. Dead cells were detected by fluorescence microscopy after staining with propidium iodide. Phase‐contrast images are also shown. MKN1 cells treated with 5μg/ml oxaliplatin (L‐OHP) for 48 h were examined as a positive control for induction of cell death. Scale bars, 500 μm

To understand the mechanism underlying the poor prognosis of gastric cancer expressing *FOLR1* at a high level, we examined the abundance of *FOLR1* mRNA and FOLRα protein in various human gastric cancer cell lines by RT‐qPCR analysis and immunoblot analysis, respectively. We found that among eight such cell lines, MKN1, NCI‐N87, and MKN74 showed relatively high levels of both *FOLR1* mRNA and FOLRα protein (Figure 2A). Immunofluorescence analysis revealed both membranous and cytoplasmic staining for FOLRα in MKN1 and MKN74 cells (Figure 2B and Supplementary Figure S1), consistent with our results for human gastric cancer specimens and supporting the validity of these cell lines for the study of FOLRα function in gastric cancer.

We next examined the effects of knockdown of *FOLR1* mRNA in MKN1 and MKN74 cells by RNA interference. The knockdown efficiency for the *FOLR1*‐targeting siRNA (siFOLR1) was found to be 78% by RT‐qPCR analysis in MKN1 cells compared to control transfected with scrambled RNA (scrambled control) (Figure 2C). Transfection with siFOLR1 attenuated cell proliferation without inducing cell death in both MKN1and MKN74 cells, although the antiproliferative effect in the latter cells did not achieve statistical significance (Figures 2D and 2E). Together, these data suggested that FOLRα is required for the proliferation but not the survival of gastric cancer cells.

### 3.3 Relation of FOLRα expression to low apoptotic activity in gastric cancer

To investigate the molecular pathways in which FOLRα functions, we performed RNA‐sequencing (RNA‐seq) analysis for tumors formed by MKN1 cells in nude mice (Figure S2A). The isolated tumor cells were first subjected to fluorescence‐activated cell sorting (FACS) to obtain two populations expressing FOLRα at a high or low level. Gene ontology (GO) analysis revealed 18 genes related to “extracellular matrix organization,” 19 genes related to ”extracellular structure organization,” 11 genes related to “regulation of extrinsic apoptotic signaling pathway,” 6 genes related to “regulation of cell adhesion mediated by integrin,” 11 genes related to “cell‐matrix adhesion,” or 6 genes related to “cell adhesion mediated by integrin” were upregulated in the FOLRα‐high population while 14 genes related “positive regulation of leukocyte proliferation,” 9 genes related to “regulation of protein activation cascade,” 13 genes related to “positive regulation of lymphocyte proliferation” were downregulated (Figure S2B).

We next performed a microarray analysis of control and FOLRα‐depleted MKN1 cells and examined the differential expression of genes related to cell proliferation, cell survival, or metastasis with the use of WikiPathways (Figure S3). Among genes related to “apoptosis,” the expression of *MDM2* was down‐regulated, whereas that of *APAF1*, *TNFSF10*, *CASP1*, *CASP6*, *CASP7*, and *PMAIP1* was up‐regulated in the FOLRα‐depleted cells. The expression of substantial numbers of genes related to “cell cycle,” “focal adhesion,” “TGF‐beta receptor signaling pathway,” or “Wnt signaling” was also downregulated by FOLRα knockdown.

Together, these findings suggested that FOLRα is positively associated with cell proliferation and negatively associated with extracellular matrix organization/adhesion and apoptosis. Given that knockdown of FOLRα suppressed cell proliferation without affecting cell viability in gastric cancer cell lines (Figure 2C–E); however, the up‐regulation of apoptosis‐related gene expression in cells depleted of FOLRα appears to be insufficient to trigger apoptosis.

### 3.4 Knockdown of FOLRα induces downregulation of MDM2

**FIGURE 3:**
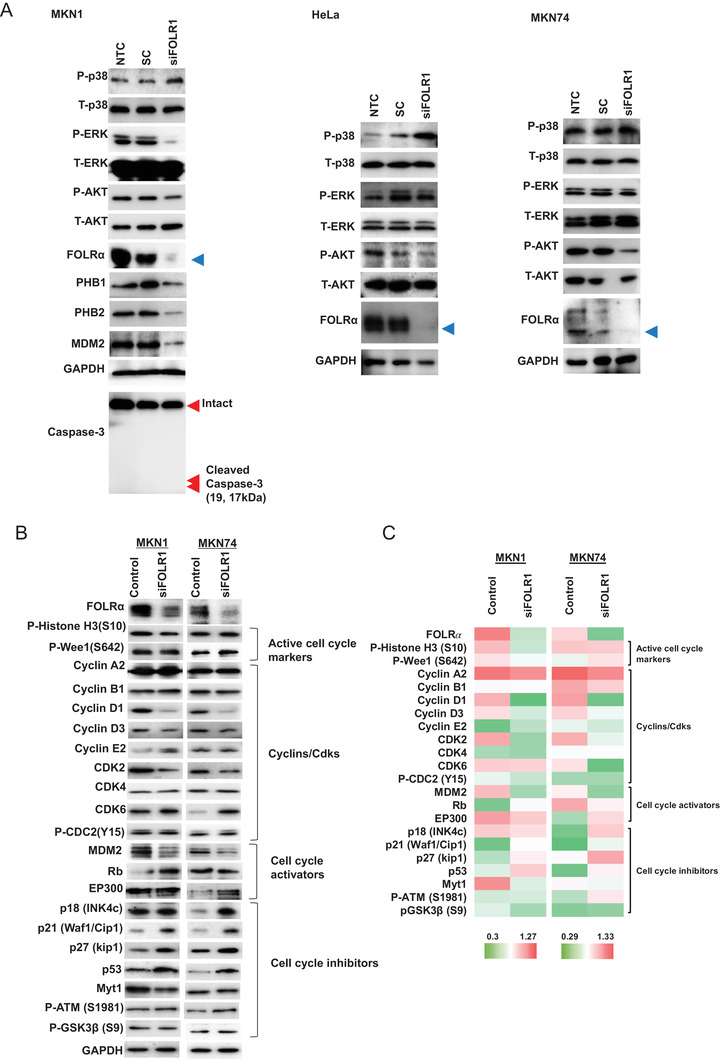
Screening of molecules that contribute to the phenotype of FOLRα‐expressing gastric cancer. A, Immunoblot analysis of the effects of FOLRα depletion on MAPK and AKT signaling in MKN1, HeLa, and MKN74 cells. Cells transfected (or not, NTC) with siFOLR1 or a scrambled control siRNA (SC) were subjected to immunoblot analysis with antibodies to the indicated proteins. Phosphorylated (P) and total (T) forms of proteins were examined, with GAPDH serving as a loading control. The blue arrowheads indicate the FOLRα bands. B, Immunoblot analysis of the effects of FOLRα knockdown on the expression of proteins related to the cell cycle or cell survival in MKN1 and MKN74 cells. MKN1 or MKN7 cells were transfected with siFOLR1 or a scrambled control siRNA. Phosphorylated residues are indicated for phosphorylated proteins. C, Heat map for the expression of proteins related to the cell cycle or cell survival in MKN1 or MKN7 cells transfected with siFOLR1 or a scrambled control siRNA. The results were derived by quantifying the immunoblot data shown in Figure 3B using ImageJ (NIH) software and GAPDH as a calibration control. Red and green correspond to high and low relative protein expression levels, respectively, in each cell line

We performed immunoblot analysis to examine the possible effects of FOLRα depletion on signaling by the mitogen‐activated protein kinases (MAPKs), ERK (extracellular signal‐regulated kinase), and p38 as well as by the protein kinase AKT (Figure 3A). However, we obtained inconsistent results for the effects of FOLRα knockdown on the phosphorylation of these proteins in MKN1 cells, MKN7 cells, and HeLa cells (a human cervical cancer cell line known to express FOLRα at a high level). To identify proteins that might interact with FOLRα and thereby contribute to the regulation of cell proliferation, we compared the scrambled control and FOLRα‐depleted MKN1 or MKN7 cells for the expression of proteins related to the cell cycle or cell survival, including active cell cycle markers (phospho–histone H3 and phospho‐Wee1), cyclins and cyclin‐dependent kinases (cyclins A2, B1, D1, D3, and E2; CDK2, CDK4, CDK6, and phospho‐CDC2), cell cycle activators (MDM2, Rb, and EP300), and cell cycle inhibitors [p18(Ink4c), p21(Waf1/Cip1), p27(Kip1), p53, Myt1, phospho‐ATM, and phospho‐GSK3β] (Figures 3B and 3C). Immunoblot analysis revealed that knockdown of FOLRα resulted in down‐regulation of cyclin D1, cyclin D3, CDK2, and MDM2 as well as in up‐regulation of p21(Waf1/Cip1), p27(Kip1), and p53 in both MKN1 and MKN74 cells (Figures 3B and 3C). Among these proteins, the ubiquitin ligase MDM2 serves as a hub that interacts with multiple proteins related to cell proliferation, cell cycle, or gene transcription.^8^, ^9^

### 3.5 PHB2 is a mediator of FOLRα‐MDM2 interaction

**FIGURE 4:**
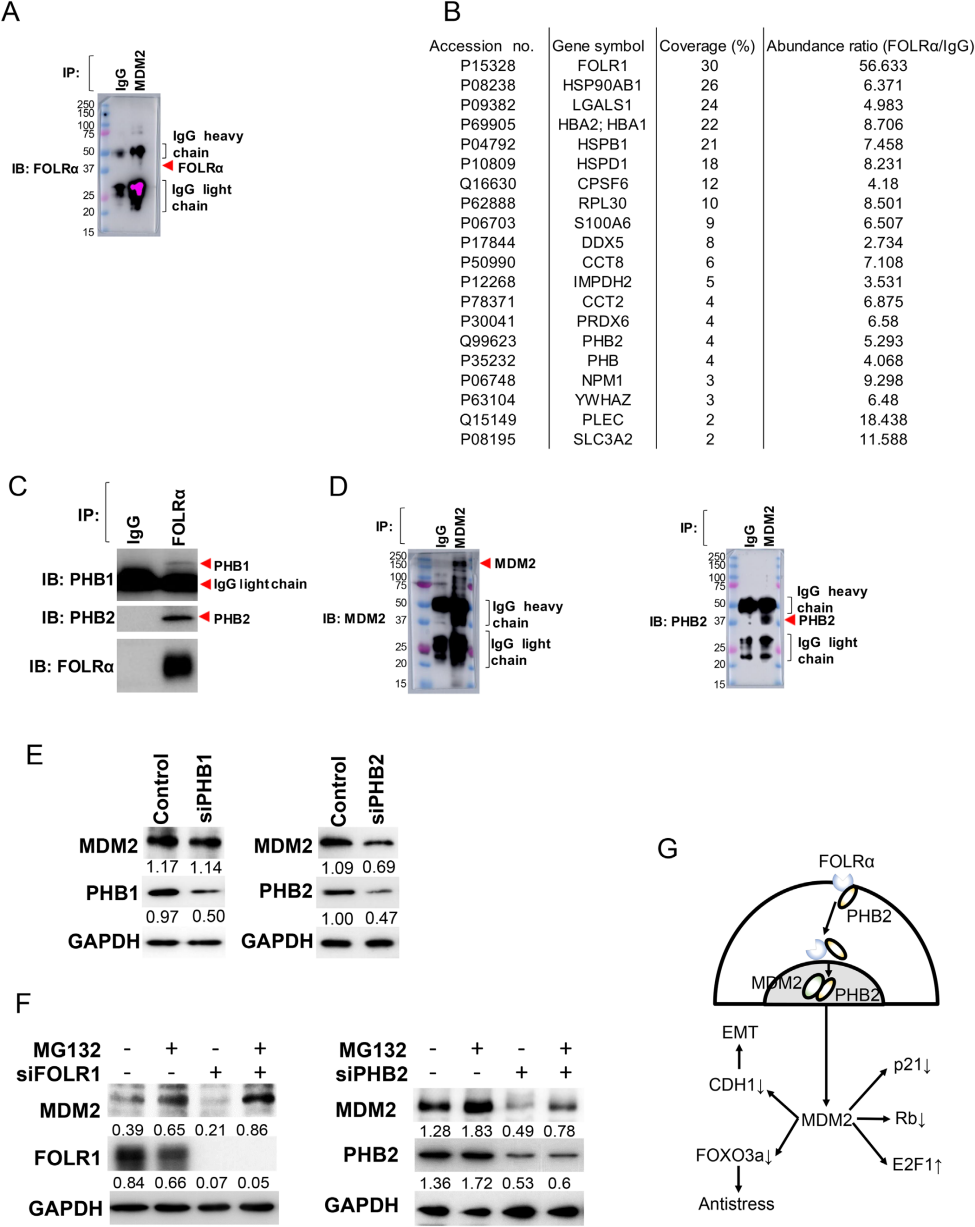
Identification of a FOLRα‐PHB2‐MDM2 axis in gastric cancer cells. A, IB analysis of FOLRα in MDM2 immunoprecipitates prepared from MKN1cells. Molecular size markers (kilodaltons) are shown in the leftmost lanes. B, Binding partners of FOLRα identified by LC‐MS/MS analysis of FOLRα immunoprecipitates prepared from HeLa cells. Among 206 such proteins identified, the 19 proteins shown in addition to FOLRα were chosen on the basis of their function and intracellular distribution. C, Immunoblot (IB) analysis of PHB1, PHB2, and FOLRα in immunoprecipitates (IP) prepared from MKN1 cells with antibodies to FOLRα or control immunoglobulin G (IgG). D, IB analysis of MDM2 and PHB2 in MDM2 immunoprecipitates prepared from MKN1cells. {Molecular size markers (kilodaltons) are shown in the leftmost lanes. E, IB analysis of MDM2, PHB1, and PHB2 in MKN1 cells transfected with siPHB1, siPHB2, or a scrambled control siRNA as indicated. Band intensity was quantified with the use of ImageJ (NIH) software and with GAPDH as a calibration control. Results are representative of three independent experiments. F, Immunoblot analysis of MDM2, FOLRα, and PHB2 in MKN1 cells transfected with siFOLR1 or siPHB2, as indicated, and then incubated in the absence or presence of MG132 at 1 nM for 6 h. Band intensity was quantified with the use of ImageJ (NIH) software and with GAPDH as a calibration control. Results are representative of three independent experiments. G, Model for the operation of a FOLRα‐PHB2‐MDM2 axis in gastric cancer. PHB2 mediates the interaction between FOLRα and MDM2 and thereby regulates p53‐dependent or ‐independent functions of MDM2, including promotion of EMT, degradation of FOXO3a, p21(Waf1/Cip1), and Rb, as well as stabilization of E2F1

We next investigated the functional relation between FOLRα and MDM2. Given that physical interaction between FOLRα and MDM2 was not detected by co‐immunoprecipitation analysis (Figure 4A), we hypothesized that another protein might mediate any such interaction between FOLRα and MDM2. To identify such a protein, we performed liquid chromatography and tandem mass spectrometry (LC‐MS/MS) of FOLRα immunoprecipitates prepared from HeLa cells (Figure 4B and Table S2). HeLa cells were used as a substitute for MKN1 cells in LC‐MS/MS analysis, given that the relative abundance of *FOLR1* mRNA in the former cells was 1.27 ± 0.32 compared with 1.03 ± 0.26 for the latter. Again, physical interaction between FOLRα and MDM2 was not detected. Among the proteins found to bind to FOLRα, we focused on those whose function is related to cell proliferation or survival and localized to the cytoplasm or cell membrane and may come into contact with FOLRα. Among such proteins identified by the LC‐MS/MS analysis, PHB2 was known to interact with MDM2 directly.^10^ We therefore further examined PHB2 and the related protein prohibitin1 (PHB1), both of which localize to mitochondria, the nucleus, and the cell membrane and contribute to the regulation of cell proliferation, apoptosis, and metastasis.^11^ Co‐immunoprecipitation analysis of MKN1 cells suggested that FOLRα might bind preferentially to PHB2 rather than to PHB1 (Figure 4C and Supplementary Figure S5). To examine whether PHB2 might mediate the interaction between FOLRα and MDM2, we performed immunoblot analysis of MDM2 immunoprecipitates prepared from MKN1 cells (Figure 4D). Whereas FOLRα was not detected in association with MDM2, PHB2 was clearly detected in the MDM2 immunoprecipitates.

We next investigated the possible effect of PHB1 or PHB2 on MDM2 expression (Figure 4E and Supplementary Figure S5). Transfection of MKN1 cells with a siRNA specific for PHB2 (siPHB2) achieved a knockdown efficiency of 53% resulted in a 37% decrease in the amount of MDM2, whereas the abundance of MDM2 was largely unchanged after transfection with a PHB1 siRNA (siPHB1), suggesting the possibility that interaction between FOLRα and PHB2 might stabilize MDM2. To determine whether FOLRα and PHB2 affect the proteasome‐mediated degradation of MDM2, we transfected MKN1 cells with siFOLR1 or siPHB2 and then exposed the transfected cells to the proteasome inhibitor MG132 (Figure 4F and Figure S5). Immunoblot analysis revealed that FOLRα or PHB2 knockdown efficiency was 89% to 94% and 56% to 69%, respectively. Whereas transfection with siFOLR1 or siPHB2 resulted in the downregulation of MDM2, MG132 increased the abundance of MDM2 both in control and in FOLRα‐ or PHB2‐depleted cells, suggesting that the proteasomal degradation of MDM2 is balanced by FOLRα‐PHB2. Given that MDM2 is implicated in epithelial‐mesenchymal transition (EMT),^12^ in degradation of the transcription factor FOXO3a,^13^ the CDK inhibitor p21(Waf1/Cip1),^14^ and the tumor suppressor Rb,^15^ as well as in the stabilization of the transcription factor E2F1,^16^ our results suggest that MDM2 might determine the malignant phenotype of FOLRα‐expressing cancer as a result of the operation of a FOLRα‐PHB2‐MDM2 axis (Figure 4G).

### 3.6 Promotion of chemotherapeutic efficacy by knockdown of FOLRα but not by farletuzumab

**FIGURE 5:**
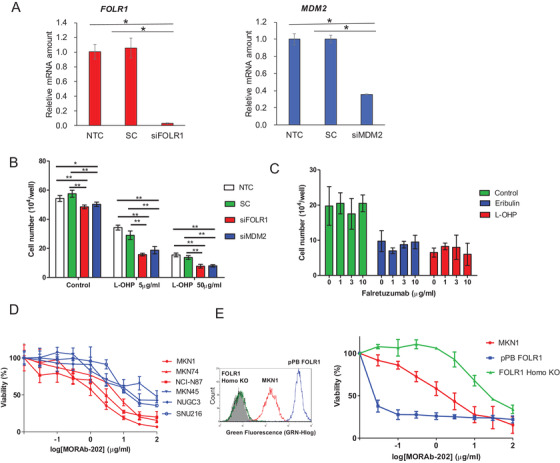
Antitumor efficacy of MORAb‐202 for FOLRα‐positive gastric cancer. A, RT‐qPCR analysis of *FOLR1* and *MDM2* mRNA abundance in MKN1 cells transfected with siFOLR1 or siMDM2, respectively. Nontransfected cells (NTC) and cells transfected with a scrambled siRNA (SC) were also examined as controls. Data are means ± SD from four independent experiments. *P < .05 (one‐way ANOVA and Tukey's post hoc test). B, Cell number for MKN1 cells transfected as in A, plated at a density of 5 × 10^4^ cells per well, and then cultured for 24 h in the absence or presence of oxaliplatin (L‐OHP, 5 or 50 μg/ml). Data are means ±SD for four independent experiments. *P < .05, **P < .01 (one‐way ANOVA followed by Tukey's post hoc test). C, Cell number for MKN1 cells plated at a density of 5 × 10^4^ cells per well, exposed to the indicated concentrations of farletuzumab for 24 h, and then incubated in the additional absence or presence of eribulin (1.5 ng/ml) or oxaliplatin (5 μg/ml) for 24 h. Data are means ± SD from four independent experiments. D, Cell proliferation assay for gastric cancer cell lines treated with various concentrations of MORAb‐202 (0.01–100 μg/ml) for 120 h. Cell lines expressing FOLRα at a high or low level are shown in red and blue, respectively. Data are means ± SD from six independent experiments. E, MKN1 cells transfected with an expression vector for human FOLRα (pPB‐FOLR1) or rendered homozygous for a disrupted *FOLR1* allele with the use of the CRISPR/Cas9 system (FOLR1 Homo‐KO) were assayed for cell surface expression of FOLRα by flow cytometry (left panel) or were incubated with various concentrations of MORAb‐202 for 120 h and then assayed for cell viability (right panel). Viability data are means ± SD from three independent experiments

Given that MDM2 is also implicated in resistance to chemotherapy,^17^, ^18^ we examined whether the FOLRα‐PHB2‐MDM2 axis might confer such resistance. We found that transfection with siFOLR1 or siMDM2 significantly inhibited the proliferation of MKN1 cells exposed to oxaliplatin (5 or 50 μg/ml), a platinum agent administered for the treatment of gastric cancer (Figures 5A and 5B). Knockdown of FOLRα thus enhanced the sensitivity of gastric cancer cells to cytotoxic chemotherapy to a similar extent as did that of MDM2.

We next examined the anticancer efficacy of the humanized anti‐FOLRα mAb farletuzumab.^19^ The addition of farletuzumab (1, 3, or 10 μg/ml) to 2 nM (1.5 ng/ml) eribulin, an anti‐microtubule agent, or to 13 μM (5 μg/ml) oxaliplatin had no additional antiproliferative effect on MKN1 cells (Figure 5C), suggestive of the limited efficacy of the addition of farletuzumab to cytotoxic agents for the treatment of gastric cancer.

### 3.7 The efficacy of MORAb‐202 depends on the expression level of FOLRα both in vitro and in vivo

We next examined the effects of a newly developed ADC, MORAb‐202, consisting of farletuzumab conjugated to eribulin. We first tested the antiproliferative activity of MORAb‐202 in human gastric cancer cell lines with various levels of FOLRα expression in vitro (Figure 5D). MORAb‐202 inhibited cell proliferation more efficiently in cell lines with a high level of FOLRα expression—including MKN1, MKN74, and NCI‐N87—than in those with a low level of FOLRα expression, including MKN45, NUGC3, and SNU216.

To confirm whether FOLRα expression indeed determines the antiproliferative activity of MORAb‐202 in vitro, we performed a cell proliferation assay with MKN1 cells in which both alleles of the endogenous *FOLR1* gene had been disrupted with the use of the CRISPR/Cas9 system (Figure S4). Indeed, we found that the efficacy of MORAb‐202 was increased in the cells overexpressing FOLRα and was attenuated in those lacking FOLRα compared with parental MKN1 cells (Figure 5E).

**FIGURE 6:**
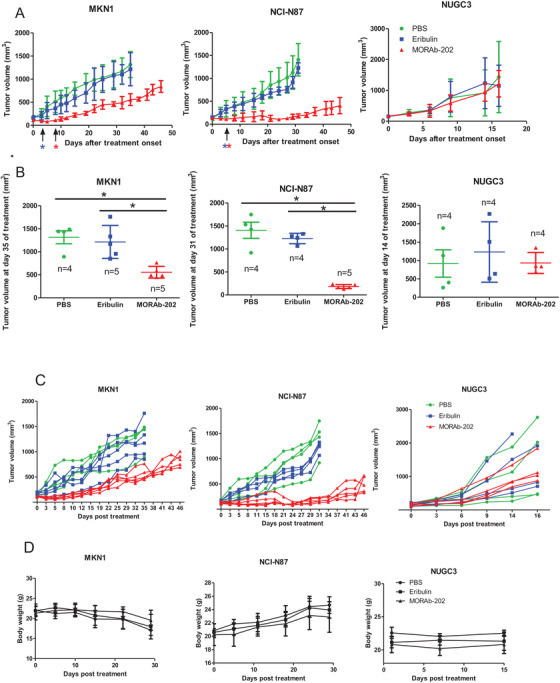
The effect of MORAb‐202 on the xenograft with high FOLRα expression and those with FOLRα low expression. A, Tumor volume for nude mice bearing subcutaneous tumors formed by MKN1, NCI‐N87, or NUGC3 cells and treated intraperitoneally with MORAb‐202 (5 mg/kg), the equivalent molar dose of eribulin (0.1 mg/kg), or PBS vehicle (100 μl) on day 0. Data are means ± SEM. Red and blue asterisks indicate *P* < .05 for comparisons between MORAb‐202 and eribulin and between MORAb‐202 and PBS at the indicated times, respectively (one‐way ANOVA followed by Tukey's post hoc test). B, Tumor volume determined as in (A) for MKN1 cells at day 35, NCI‐N87 cells at day 31, and NUGC3 cells at day 14. Individual values and the mean ± SEM are shown. *P < .05. C, Tumor volume for each mouse treated with MORAb‐202, eribulin, or PBS in Figure 6A. D, The body weight of mice treated with MORAb‐202, eribulin, or PBS in Figure 6A. Data in D are means ± SEM

**FIGURE 7:**
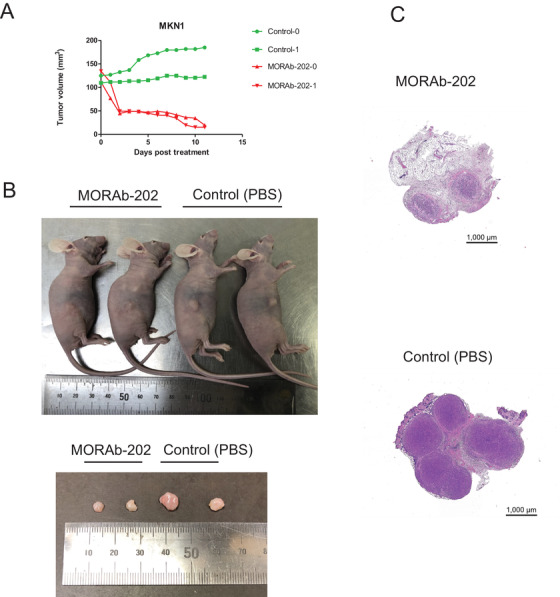
Inhibition of tumor growth by MORAb‐202 in nude mice xenografted with MKN1. A, Tumor volume of each mouse treated with MORAb‐202 or PBS. B, Photographs of mice and dissected tumors on day11. C, Representative images of hematoxylin and eosin‐stained sections of tumors from mice treated with MORAb‐202 and mice treated with PBS (Control)

Finally, we compared the antitumor activity of MORAb‐202 and an equivalent molar dose of eribulin in nude mice harboring tumors formed by gastric cancer cell lines expressing FOLRα at a high (MKN1, NCI‐N87) or low (NUGC3) level. Consistent with previous findings,^6^ MORAb‐202 had a marked and prolonged inhibitory effect on the growth of tumors formed by MKN1 or NCI‐N87 cells (Figure 6A–C). MORAb‐202 at a dose of 5 mg/kg showed a significantly (*P* < .05) higher antitumor efficacy than did the equivalent molar dose of eribulin in MKN1 cells from day 8 and in NCI‐N87 cells from day 5. MORAb‐202 at this dose had no effect on body weight in the treated mice (Figure 6D). In contrast, MORAb‐202 did not affect the growth of NUGC3 xenografts (Figure 6A–C). Antitumor activity of MORAb‐202 was observed on day 11 (Figure 7A–C). Together, our results revealed target‐specific antitumor activity of MORAb‐202 for gastric cancer cells expressing FOLRα at a high level.

## 4 DISCUSSION

We found that FOLRα is expressed in various histological subtypes of gastric cancer, with this expression being more frequent in HER2‐positive cases than in HER2‐negative ones, as previously shown for *EGFR* mutation‐positive lung adenocarcinoma,^20^ suggestive of an association between FOLRα expression and an aggressive phenotype of cancer. RNA‐seq analysis of FOLRα‐high and FOLRα‐low fractions revealed lower levels of expression of apoptosis‐related genes and higher levels of protein activation cascade– and proliferation‐related genes in the FOLRα‐high cells and downregulation of FOLRα affected cell proliferation. Furthermore, ablation of the *FOLR1* gene by CRISPR/Cas9 technology did not seem to affect MKN1 cell survival. These results thus indicate that FOLRα is not essential for the survival of gastric cancer cells but contributes to the regulation of cell proliferation under basal conditions.

We found that FOLRα was localized not only at the cell membrane and but also in the cytoplasm both of gastric cancer cells in human tumor specimens and of MKN1 and MKN74 cell lines, suggesting that FOLRα might interact with cytoplasmic proteins such as protein kinases. Although FOLRα was previously shown to contribute to the regulation of the ERK signaling pathway,^4^ we did not detect consistent effects of FOLRα knockdown on signaling by ERK, p38 MAPK, or AKT, suggesting that the interaction between FOLRα and ERK may be dependent on cell type or context. To identify molecules that contribute to the phenotype of FOLRα‐expressing gastric cancer, we performed immunoblot and microarray analyses focusing on molecules related to cell proliferation or survival, and we found that knockdown of FOLRα resulted in down‐regulation of MDM2 expression.

MDM2 is a key player in regulating cell proliferation, TGF‐β signaling,^21^ cell adhesion and invasion, and EMT,^12^ and it contributes to chemotherapy resistance.^17^, ^18^ It was, therefore, a candidate mediator of the link between FOLRα expression and malignancy of gastric cancer. However, the co‐immunoprecipitation analysis did not detect a substantial physical association between FOLRα and MDM2, suggesting that another molecule might be required to mediate the interaction between these two proteins. We performed LC‐MS/MS analysis of FOLRα immunoprecipitates to identify such a molecule. We focused on PHB1 and PHB2, which together function as a chaperone and stabilize various proteins in mitochondria.^11^, ^22^ We found that PHB2 bound to MDM2 in a co‐immunoprecipitation assay, consistent with the previous finding that PHB2 can bind to MDM2 through its p53 BOX‐I binding site,^10^ and that knockdown of PHB2 resulted in down‐regulation of MDM2 expression. On the basis of these findings, we concluded that FOLRα interacts with MDM2 via PHB2 and that such interaction mediates the stabilization of MDM2. Thus, our results identified the operation of a FOLRα‐PHB2‐MDM2 axis in gastric cancer cells, and they further implicated this axis in chemotherapy resistance.

We thus found that knockdown of FOLRα, as well as that of MDM2, significantly increased the sensitivity of MKN1 cells to oxaliplatin in vitro. We, therefore, examined the effect of farletuzumab, a humanized mAb to FOLRα that has shown antitumor efficacy in a preclinical model of ovarian cancer.^19^, ^23^ In MNK1 cells, however, farletuzumab did not show antitumor efficacy either as monotherapy or in combination with oxaliplatin or eribulin. This difference between the effects of FOLRα knockdown and farletuzumab may be attributable to the fact that the mAb simply binds to the extracellular domain of FOLRα, and it might therefore not affect the operation of the FOLRα‐PHB2‐MDM2 axis in the cytoplasm. Thus our results suggest that targeting of FOLRα alone by farletuzumab may not be sufficient to downregulate MDM2 function in gastric cancer and that the therapeutic efficacy of farletuzumab, even in combination with chemotherapy, may be limited.

We next evaluated the efficacy of MORAb‐202, a farletuzumab‐eribulin ADC that is currently under clinical investigation in gastric cancer cell lines. Consistent with previous findings in other tumor types,^6^ we found that the antitumor efficacy of MORAb‐202 for gastric cancer cell lines in vitro and in vivo was dependent on the level of FOLRα expression and that that for tumors formed by FOLRα‐high cell lines in vivo was greater than the efficacy of an equivalent dose of eribulin. Our results thus provide a rationale for the therapeutic targeting of FOLRα‐positive gastric cancer with MORAb‐202.

A limitation of the present study is that we evaluated only MORAb‐202 as a FOLRα‐targeted ADC and eribulin as a payload. Given that eribulin has been shown to suppress EMT,^24^ it is expected to be effective against cancers with a phenotype characterized by down‐regulation of the expression of genes related to cell‐cell or cell‐matrix adhesion. A clinical trial of MORAb‐202 in patients with FOLRα‐positive solid cancers is ongoing and has revealed encouraging antitumor activity (NCT03386942). Support for the treatment of FOLRα‐positive tumors with a FOLRα‐targeted ADC would be provided by elucidating the underlying mechanism of action. Here we provide a rationale for patient selection for treatment with MORAb‐202 on the basis of immunohistochemical determination of FOLRα expression in a tumor specimen. Given the substantial prevalence of FOLRα expression in gastric cancer, our data support the clinical development of MORAb‐202 for this tumor type.

## AUTHOR CONTRIBUTIONS

Hitomi Sakai was associated with conceptualization, methodology, data acquisition, data analysis, and writing the original draft. Hisato Kawakami was associated with conceptualization, methodology, data acquisition, data analysis, and revision of the manuscript. Takeshi Teramura was associated with conceptualization, methodology, data acquisition, data analysis, and revision of the manuscript. Yuta Onodera was associated with methodology, data acquisition, and data analysis. Elizabeth Somers was associated with methodology, data acquisition, and revision of the manuscript. Keiji Furuuchi was associated with data acquisition and material support. Toshimitsu Uenaka was associated with data acquisition and material support. Ryoji Kato was associated with methodology, data analysis, and revision of the manuscript. Kazuhiko Nakagawa was associated with conceptualization and study supervision. All the authors have final approval of the submitted paper.

## ETHICS APPROVAL AND CONSENT TO PARTICIPATE

All animal experiments were approved by the Animal Ethics Committee of Kindai University.

## CONSENT FOR PUBLICATION

This manuscript has been read and approved by all the authors to publish and is not submitted or under consideration for publication elsewhere.

## CONFLICT OF INTEREST

HS has received research funding from Eisai Co. Ltd and Chugai Pharmaceutical Co. Ltd. HK has received consulting fees from Bristol‐Myers Squibb Co. Ltd., Eli Lilly Japan K.K., MSD K.K., Ono Pharmaceutical Co. Ltd., Daiichi‐Sankyo Co. Ltd., and Taiho Pharmaceutical Co. Ltd; honoraria from Bristol‐Myers Squibb Co. Ltd., AstraZeneca K.K., Bayer yakuhin Ltd., Eli Lilly Japan K.K., MSD K.K., Ono Pharmaceutical Co. Ltd., Chugai Pharmaceutical Co. Ltd., Daiichi‐Sankyo Co. Ltd., Takeda Pharmaceutical Co. Ltd., and Taiho Pharmaceutical Co. Ltd.; lecture fees from Bristol‐Myers Squibb Co. Ltd., Eli Lilly Japan K.K., MSD K.K., Ono Pharmaceutical Co. Ltd., Chugai Pharmaceutical Co. Ltd., Takeda Pharmaceutical Co. Ltd., and Taiho Pharmaceutical Co. Ltd.; and research funding from Chugai Pharmaceutical Co. Ltd., Taiho Pharmaceutical Co. Ltd, and Eisai Co. Ltd. KN has received grants and personal fees from AstraZeneca K.K., grants and personal fees from Astellas Pharma Inc., grants and personal fees from MSD K.K., grants, personal fees and other from Ono Pharmaceutical Co.,Ltd., grants and personal fees from Nippon Boehringer Ingelheim Co.,Ltd., grants and personal fees from Novartis Pharma K.K., grants, personal fees and other from Pfizer Japan Inc., grants and personal fees from Bristol Myers Squibb Company, grants, personal fees and other from Eli Lilly Japan K.K., grants and personal fees from Chugai Pharmaceutical Co.,Ltd., grants and personal fees from Daiichi Sankyo Co., Ltd., grants and personal fees from Merck Serono Co., Ltd./ Merck Biopharma Co., Ltd., during the conduct of the study; personal fees from Clinical Trial Co., Ltd., personal fees from MEDICUS SHUPPAN,Publishers Co., Ltd., personal fees from Care Net, Inc, personal fees from Reno. Medical K.K., personal fees and other from KYORIN Pharmaceutical Co.,Ltd., personal fees from Medical Review Co., Ltd., personal fees from Roche Diagnostics K.K., personal fees from Bayer Yakuhin, Ltd, personal fees from Medical Mobile Communications co., Ltd, personal fees from 3H Clinical Trial Inc., personal fees from Nichi‐Iko Pharmaceutical Co., Ltd., grants, personal fees and other from Takeda Pharmaceutical Co.,Ltd., grants and personal fees from Taiho Pharmaceutical Co.,Ltd., grants and personal fees from SymBio Pharmaceuticals Limited., personal fees from NANZANDO Co.,Ltd., personal fees from YODOSHA CO., LTD., personal fees from Nikkei Business Publications, Inc, personal fees from Thermo Fisher Scientific K.K., personal fees from YOMIURI TELECASTING CORPORATION., personal fees from Nippon Kayaku Co.,Ltd., grants and personal fees from AbbVie Inc, grants from inVentiv Health Japan, grants from ICON Japan K.K., grants from GRITSONE ONCOLOGY.INC, grants from PAREXEL International Corp., grants from Kissei Pharmaceutical Co.,Ltd., grants from EPS Corporation., grants from Syneos Health., grants from Pfizer R&D Japan G.K., grants from A2 Healthcare Corp., grants from Quintiles Inc. / IQVIA Services JAPAN K.K., grants from EP‐CRSU CO., LTD., grants from Linical Co.,Ltd., grants from Eisai Co., Ltd., grants from CMIC Shift Zero K.K., grants from Kyowa Hakko Kirin Co.,Ltd, grants from Bayer Yakuhin, Ltd, grants from EPS International Co.,Ltd,., grants from Otsuka Pharmaceutical Co., Ltd., outside the submitted work; . RK has received lecture fees from Bristol‐Myers Squibb Co. Ltd.; and honoraria from Eli Lilly Japan K.K. K. E. Somers, K. Furuuchi, and T. Uenaka are employees of Eisai Inc. TT and YO declare no potential conflicts of interest.

## Supporting information

Supporting Information

Supplementary Figure S1. Confocal micrographs showing immunofluorescence staining for FOLRα in MKN1. Cells were incubated with MORAb‐003 and Polyclonal Rabbit Anti‐Human IgG/FITC (DAKO, F020202). Nikon laser scanning microscope (Eclipse Ti equipped with Nikon C2 Si laser scanning unit) and imaged with an x40 oil immersion objective lens.
Supplementary Figure S2. RNA‐seq analysis of MKN1 tumor cells expressing FOLRα at a high or low level in a mouse xenograft model. A, Tumors formed in BALB/c nude mice at 20 days after subcutaneous injection of MKN1 cells (1 × 10^7^) were removed to fractionate tumor cells expressing FOLRα at a high or low level by FACS. Total RNA extracted from each cell population was then subjected to RNA‐seq analysis. B, GO enrichment analysis of differentially expressed genes in the FOLRα‐high tumor cells relative to the FOLRα‐low cells. The numbers of genes for each term among the 182 down‐regulated genes (green) or the 276 up‐regulated genes (red) in the FOLRα‐high cells are indicated. Terms highlighted in blue are related to cell survival, proliferation, or metastasis.
Supplementary Figure S3. Microarray analysis of gene expression in control and FOLRα‐depleted MKN1 cells. Genes whose expression was significantly up‐regulated or down‐regulated in MKN1 cells transfected with siFOLR1 in comparison with those transfected with a scrambled control siRNA are grouped according to WikiPathways. *N* indicates the number of affected genes among the total number of genes in each pathway.
Supplementary Figure S4. CRISPR/Cas9‐mediated knockout (KO) of *FOLR1* in MKN1 cells. A, The design of guide RNA. B, Genomic PCR for genotyping of colonies. C, Successful targeting of *FOLR1* was confirmed by DNA sequencing.

Supplementary Figure S5. Raw images of Figure3D, 3F, and 3G. After protein transfer, the membranes, including objective proteins, were cut out and used for subsequent reactions. Green arrows indicate the lanes of objective proteins, and red arrows represent the molecular weight of target proteins. Image analyzers used are shown on the left of the membrane. The image analyzer used is presented to the left of the membrane.

Supplementary Table S1. Summary of FOLRα positivity in different histological subtypes of gastric cancer.

Supplementary Table S2. List of binding partners of FOLRα identified by LC‐MS/MS analysis of FOLRα immunoprecipitates prepared from HeLa cells.

## Data Availability

All data that support the findings of this study are available from the corresponding author upon reasonable request.
