# Supplementary material for: Folate receptor α increases chemotherapy resistance through stabilizing MDM2 in cooperation with PHB2 that is overcome by MORAb‐202 in gastric cancer
Source: Clin Transl Med. 2021 Jun 1;11(6):e454. doi: 10.1002/ctm2.454 (PMC8167866; doi:10.1002/ctm2.454)
Supplement: Supplementary file 1 — Supporting Information [file CTM2-11-e454-s005.docx]

**SUPPLEMENTARY METHODS**

**Tissue microarray analysis**

Gastric Cancer TMA ST1401(USBioMax) was used for analysis. ST1401 is a stomach adenocarcinoma tissue microarray, containing 139 cases of adenocarcinoma and 1 stomach tissue, IHC results shows 88 cases adenocarcinoma Her-2 (0, 1+), 39 adenocarcinoma Her-2 (3+), 10 adenocarcinoma Her-2 (2+) and 1 Her-2 (1+) stomach tissue, single core per case, the TMA slides were baked at 60 degrees for one hour prior to deparaffinization in xylene and graded alcohol baths. HIER-antigen retrieval was performed using Diva Decloaker solution at 95 degrees for 40minutes. After cooling to room temperature, slides were washed 3 times in TBST. Peroxidase and protein blocking steps were followed by 30 minute incubations with the primary antibodies (either anti-folate receptor alpha antibody 26B3 (1ug/mL) or a negative control mouse IgG antibody). After washing slides were developed using the MACH4 Mouse HRP-Polymer Detection Kit and counterstained with hematoxylin.

**Fluorescence-activated cell sorting (FACS)**

Standard protocols were followed for all experiments. Tumor xenografts were treated with trypsin to obtain a single-cell suspension, and the isolated cells were then resuspended in Dulbecco’s modified Eagle’s medium (DMEM) and incubated with antibodies to FOLRα for 60 min at 4°C. Unbound primary antibodies were removed by washing the cells with DMEM. The cells were then incubated with the secondary antibodies for 60 min at 4°C (BD Biosciences).

FACS Aria (BD Biosciences) was used for cell sorting.

**RNA sequencing (RNA-seq)**

FOLRα-high and FOLRα-low cells were isolated from MKN1 tumor xenografts by FACS. Total RNA was isolated from the sorted cells with the use of the TRIzol reagent and was used for library preparation with the use of a SMART-seq Stranded Kit (Takara Bio). Next-generation RNA sequencing was performed with a NextSeq 500 instrument (Illumina) set in the paired-end 50 + 25 bp high-output sequencing mode. The trimmed reads were mapped to the reference human genome (GRCh38) with the STAR program (version 2.6.1a). Sequence quality was assessed with FastQC software. Gene Ontology analysis was performed using the clusterProfiler software developed by Yu G et al. (“clusterProfiler: an R package for comparing biological themes among gene clusters.” OMICS, 16(5): 284-287).

**Microarray Analysis**

The microarray gene expression analysis of MKN1 transfected with siFOLR1 and scrambled control were performed using the Clariom S Human Array (Thermo Fisher Scientific) and the Affymetrix Transcriptome Analysis Console software (Thermo Fisher　Scientific)，following the standard protocol.
