# Supplementary material for: Folate receptor α increases chemotherapy resistance through stabilizing MDM2 in cooperation with PHB2 that is overcome by MORAb‐202 in gastric cancer
Source: Clin Transl Med. 2021 Jun 1;11(6):e454. doi: 10.1002/ctm2.454 (PMC8167866; doi:10.1002/ctm2.454)
Supplement: Supplementary file 2 — Supplementary Figure S1. Confocal micrographs showing immunofluorescence staining for FOLRα in MKN1. Cells were incubated with MORAb‐003 and Polyclonal Rabbit Anti‐Human IgG/FITC (DAKO, F020202). Nikon laser scanning microscope (Eclipse Ti equipped with Nikon C2 Si laser scanning unit) and imaged with an x40 oil immersion objective lens. Supplementary Figure S2. RNA‐seq analysis of MKN1 tumor cells expressing FOLRα at a high or low level in a mouse xenograft model. A, Tumors formed in BALB/c nude mice at 20 days after subcutaneous injection of MKN1 cells (1 × 10 7 ) were removed to fractionate tumor cells expressing FOLRα at a high or low level by FACS. Total RNA extracted from each cell population was then subjected to RNA‐seq analysis. B, GO enrichment analysis of differentially expressed genes in the FOLRα‐high tumor cells relative to the FOLRα‐low cells. The numbers of genes for each term among the 182 down‐regulated genes (green) or the 276 up‐regulated genes (red) in the FOLRα‐high cells are indicated. Terms highlighted in blue are related to cell survival, proliferation, or metastasis. Supplementary Figure S3. Microarray analysis of gene expression in control and FOLRα‐depleted MKN1 cells. Genes whose expression was significantly up‐regulated or down‐regulated in MKN1 cells transfected with siFOLR1 in comparison with those transfected with a scrambled control siRNA are grouped according to WikiPathways. N indicates the number of affected genes among the total number of genes in each pathway. Supplementary Figure S4. CRISPR/Cas9‐mediated knockout (KO) of FOLR1 in MKN1 cells. A, The design of guide RNA. B, Genomic PCR for genotyping of colonies. C, Successful targeting of FOLR1 was confirmed by DNA sequencing. [file CTM2-11-e454-s004.pptx]

## Slide 1
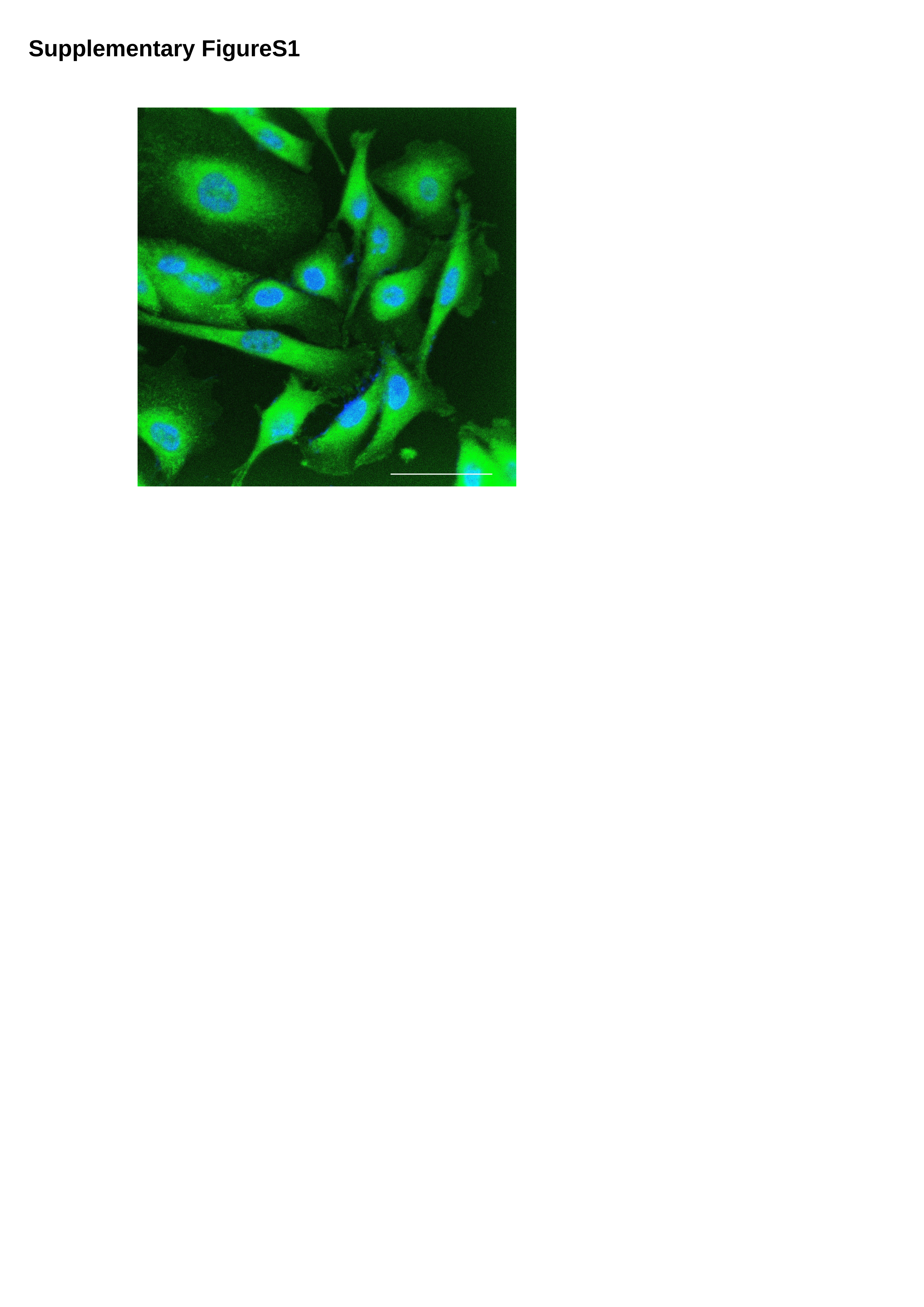

Supplementary FigureS1

## Slide 2
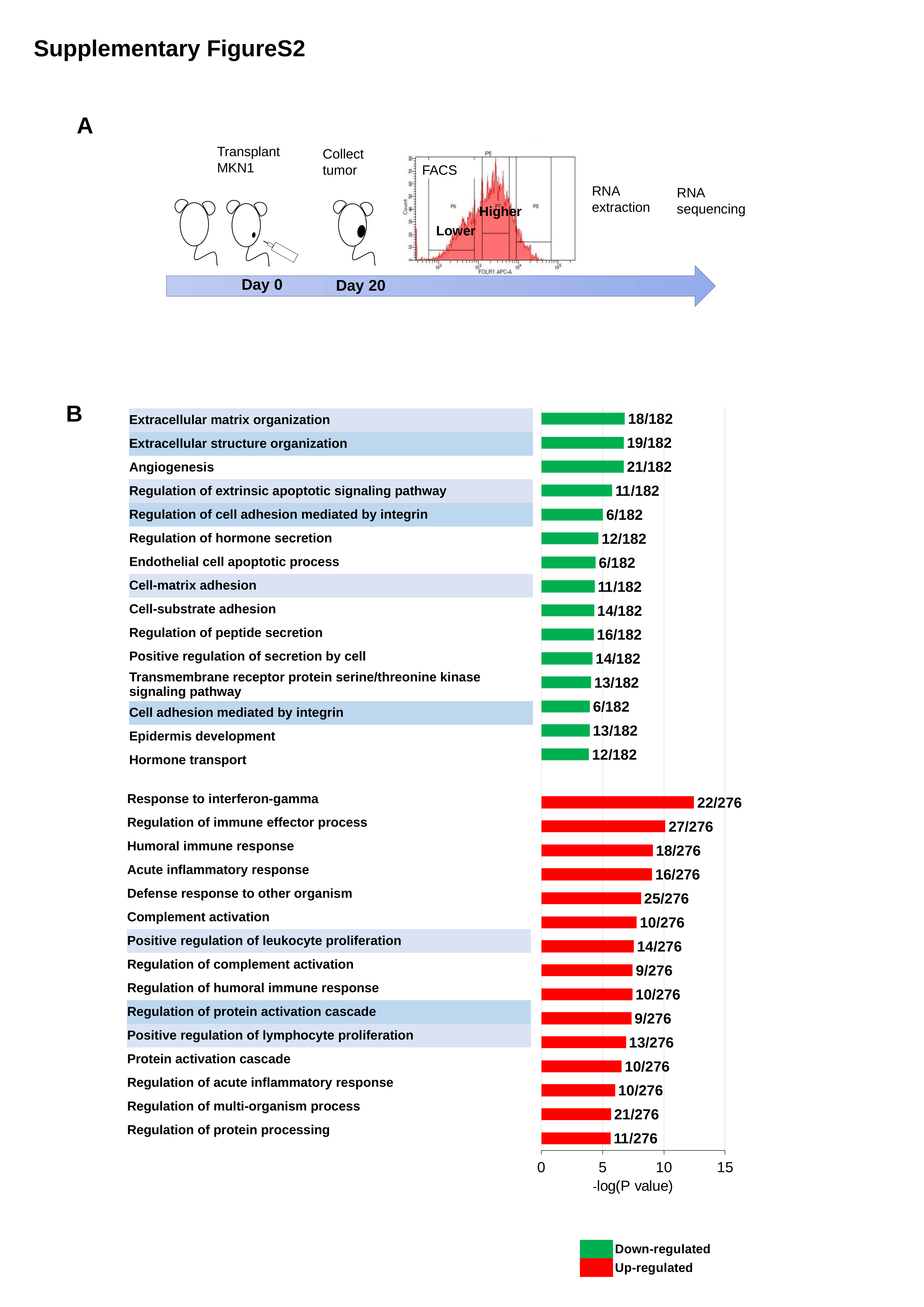

Supplementary FigureS2
A
Transplant
MKN1
Collect
tumor
FACS
RNA
extraction
RNA
sequencing
Higher
Lower
Day 0
Day 20
B
| Extracellular matrix organization |
| --- |
| Extracellular structure organization |
| Angiogenesis |
| Regulation of extrinsic apoptotic signaling pathway |
| Regulation of cell adhesion mediated by integrin |
| Regulation of hormone secretion |
| Endothelial cell apoptotic process |
| Cell-matrix adhesion |
| Cell-substrate adhesion |
| Regulation of peptide secretion |
| Positive regulation of secretion by cell |
| Transmembrane receptor protein serine/threonine kinase signaling pathway |
| Cell adhesion mediated by integrin |
| Epidermis development |
| Hormone transport |
| Response to interferon-gamma |
| --- |
| Regulation of immune effector process |
| Humoral immune response |
| Acute inflammatory response |
| Defense response to other organism |
| Complement activation |
| Positive regulation of leukocyte proliferation |
| Regulation of complement activation |
| Regulation of humoral immune response |
| Regulation of protein activation cascade |
| Positive regulation of lymphocyte proliferation |
| Protein activation cascade |
| Regulation of acute inflammatory response |
| Regulation of multi-organism process |
| Regulation of protein processing |

## Slide 3
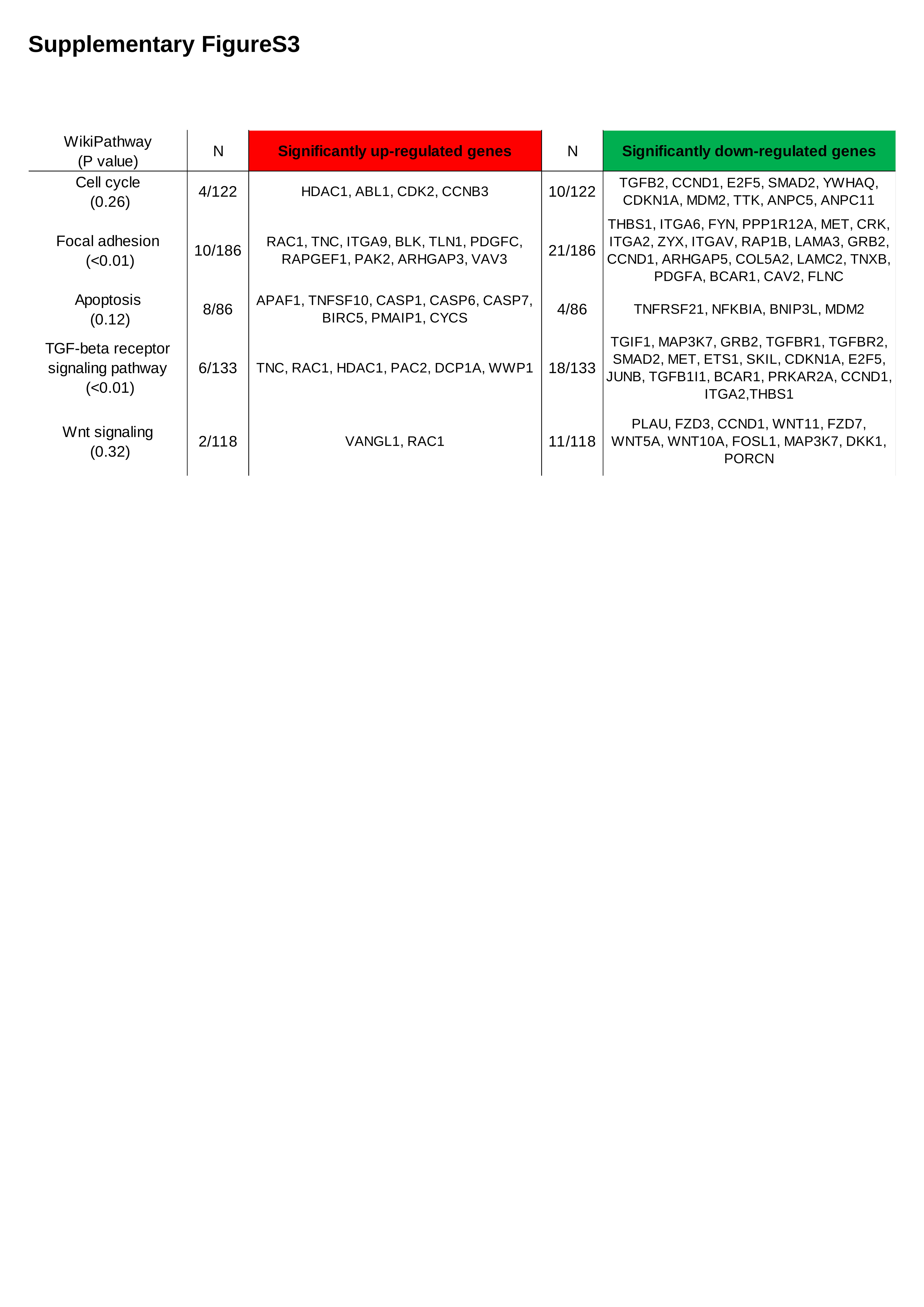

Supplementary FigureS3

## Slide 4
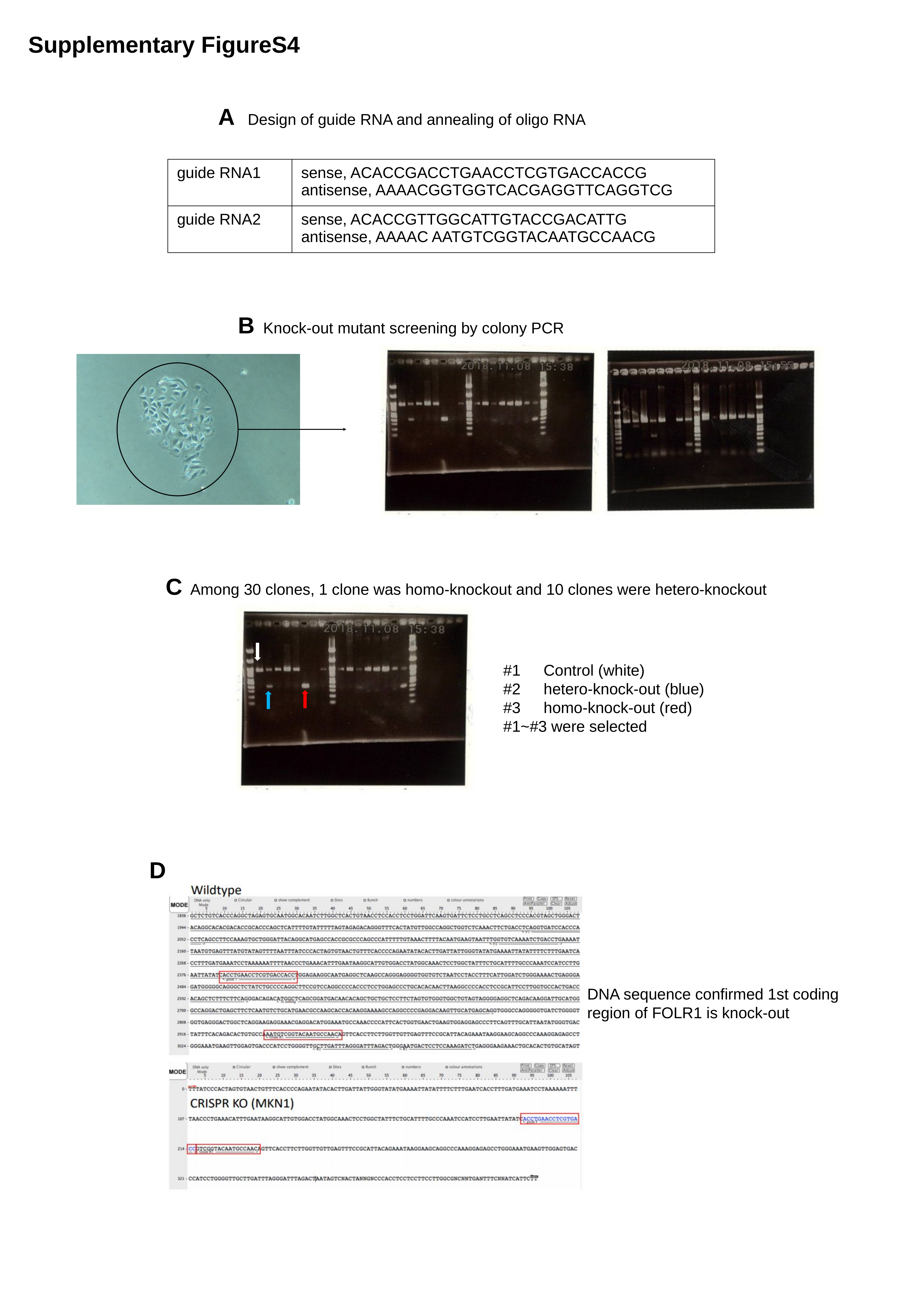

Supplementary FigureS4
A Design of guide RNA and annealing of oligo RNA
| guide RNA1 | sense, ACACCGACCTGAACCTCGTGACCACCG antisense, AAAACGGTGGTCACGAGGTTCAGGTCG |
| --- | --- |
| guide RNA2 | sense, ACACCGTTGGCATTGTACCGACATTG antisense, AAAAC AATGTCGGTACAATGCCAACG |
B Knock-out mutant screening by colony PCR
C Among 30 clones, 1 clone was homo-knockout and 10 clones were hetero-knockout
#1　Control (white)
#2　hetero-knock-out (blue)
#3　homo-knock-out (red)
#1~#3 were selected
D
DNA sequence confirmed 1st coding region of FOLR1 is knock-out
