# Supplementary material for: Folate receptor α increases chemotherapy resistance through stabilizing MDM2 in cooperation with PHB2 that is overcome by MORAb‐202 in gastric cancer
Source: Clin Transl Med. 2021 Jun 1;11(6):e454. doi: 10.1002/ctm2.454 (PMC8167866; doi:10.1002/ctm2.454)
Supplement: Supplementary file 3 — Supplementary Figure S5. Raw images of Figure3D, 3F, and 3G. After protein transfer, the membranes, including objective proteins, were cut out and used for subsequent reactions. Green arrows indicate the lanes of objective proteins, and red arrows represent the molecular weight of target proteins. Image analyzers used are shown on the left of the membrane. The image analyzer used is presented to the left of the membrane. [file CTM2-11-e454-s001.pptx]

## Slide 1
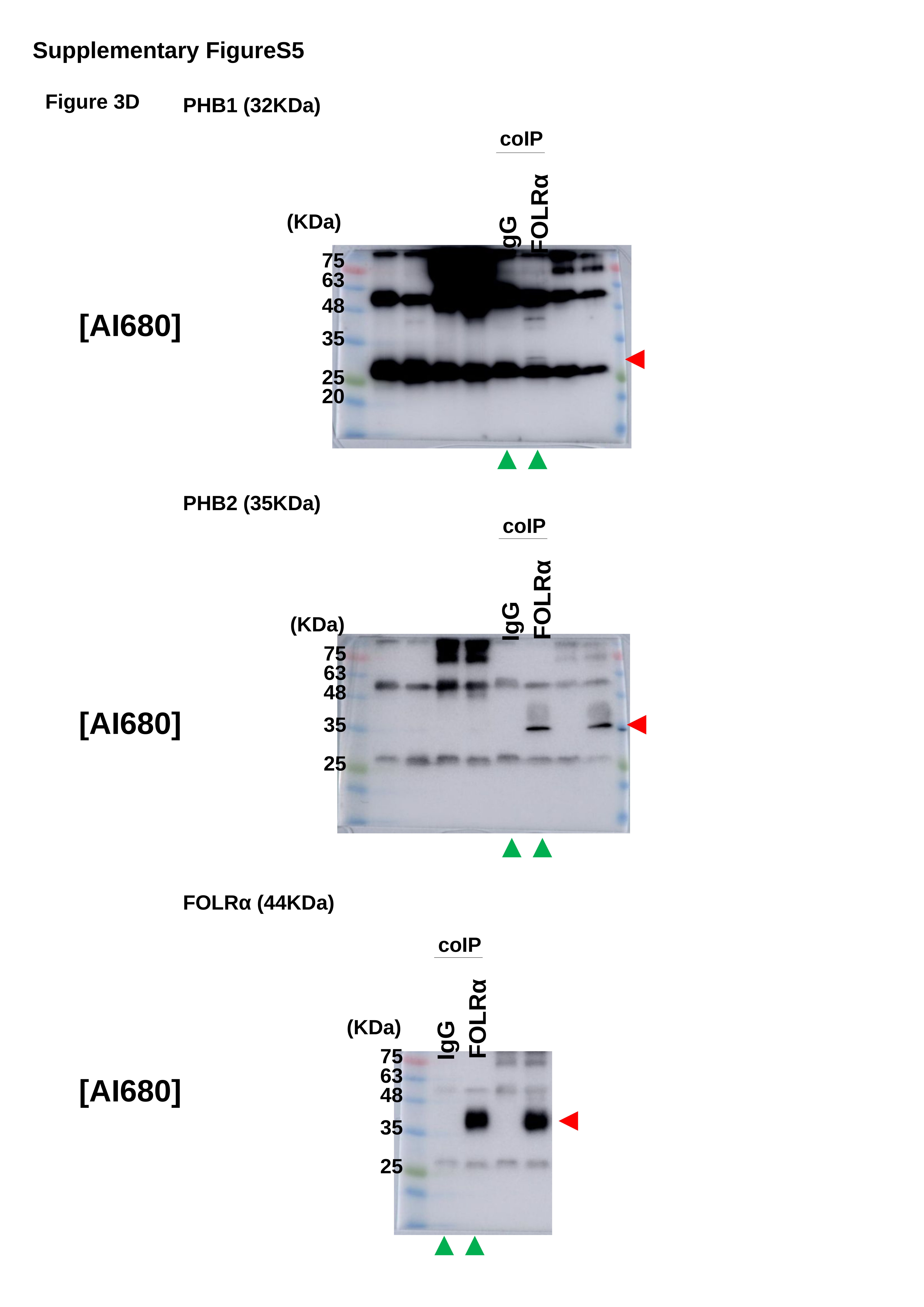

Supplementary FigureS5
Figure 3D
PHB1 (32KDa)
coIP
FOLRα
IgG
(KDa)
75
63
48
35
25
20
[AI680]
PHB2 (35KDa)
coIP
FOLRα
IgG
(KDa)
75
63
48
35
25
[AI680]
FOLRα (44KDa)
coIP
FOLRα
IgG
(KDa)
75
63
48
35
25
[AI680]

## Slide 2
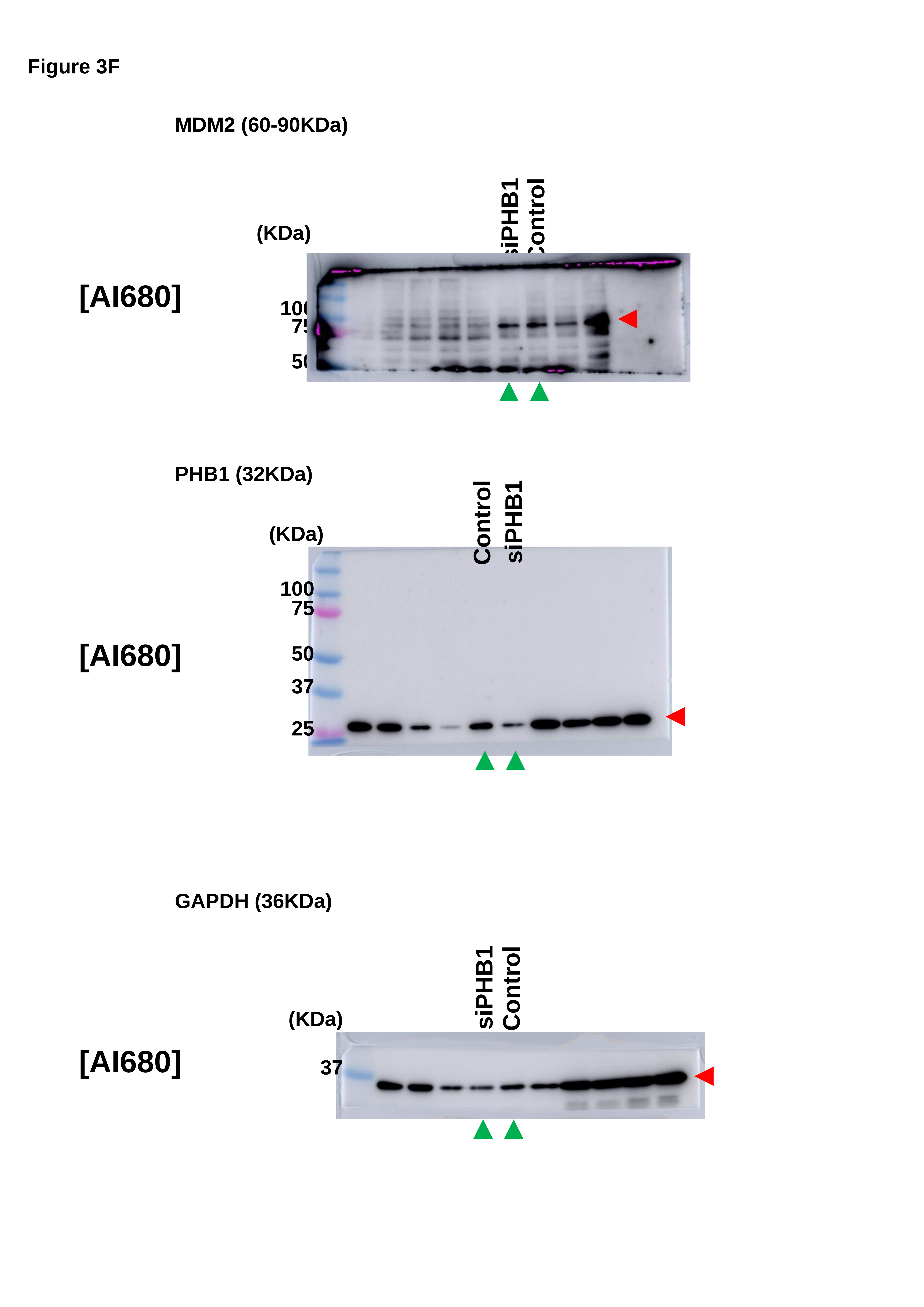

Figure 3F
MDM2 (60-90KDa)
siPHB1
Control
(KDa)
100
75
50
[AI680]
PHB1 (32KDa)
siPHB1
Control
(KDa)
100
75
50
37
25
[AI680]
GAPDH (36KDa)
siPHB1
Control
(KDa)
37
[AI680]

## Slide 3
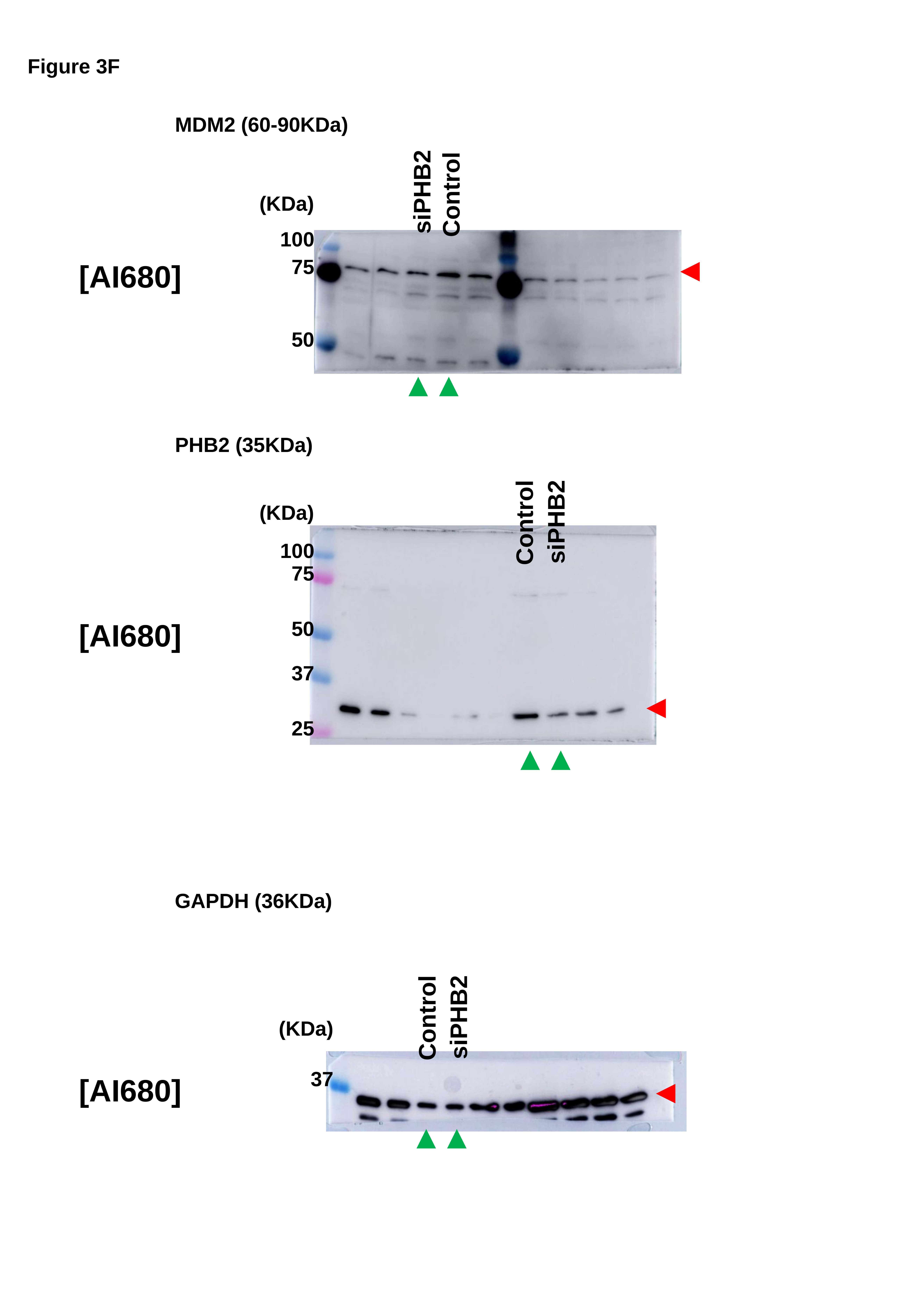

Figure 3F
MDM2 (60-90KDa)
siPHB2
Control
(KDa)
100
75
50
[AI680]
PHB2 (35KDa)
siPHB2
Control
(KDa)
100
75
50
37
25
[AI680]
GAPDH (36KDa)
siPHB2
Control
(KDa)
37
[AI680]

## Slide 4
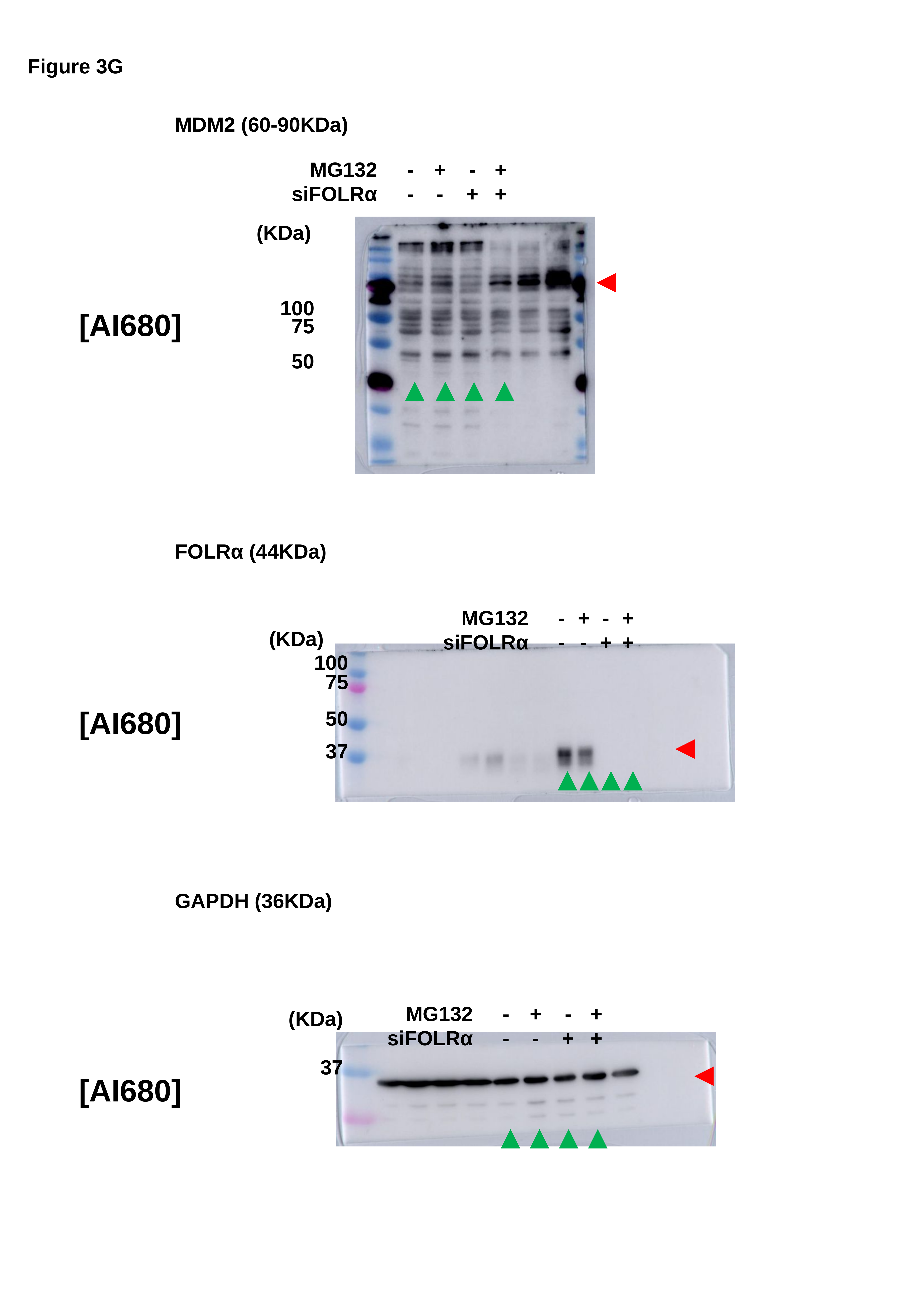

Figure 3G
MDM2 (60-90KDa)
MG132
-
+
-
+
siFOLRα
-
-
+
+
(KDa)
100
75
50
[AI680]
FOLRα (44KDa)
MG132
-
+
-
+
(KDa)
siFOLRα
-
-
+
+
100
75
50
37
[AI680]
GAPDH (36KDa)
MG132
-
+
-
+
siFOLRα
-
-
+
+
(KDa)
37
[AI680]

## Slide 5
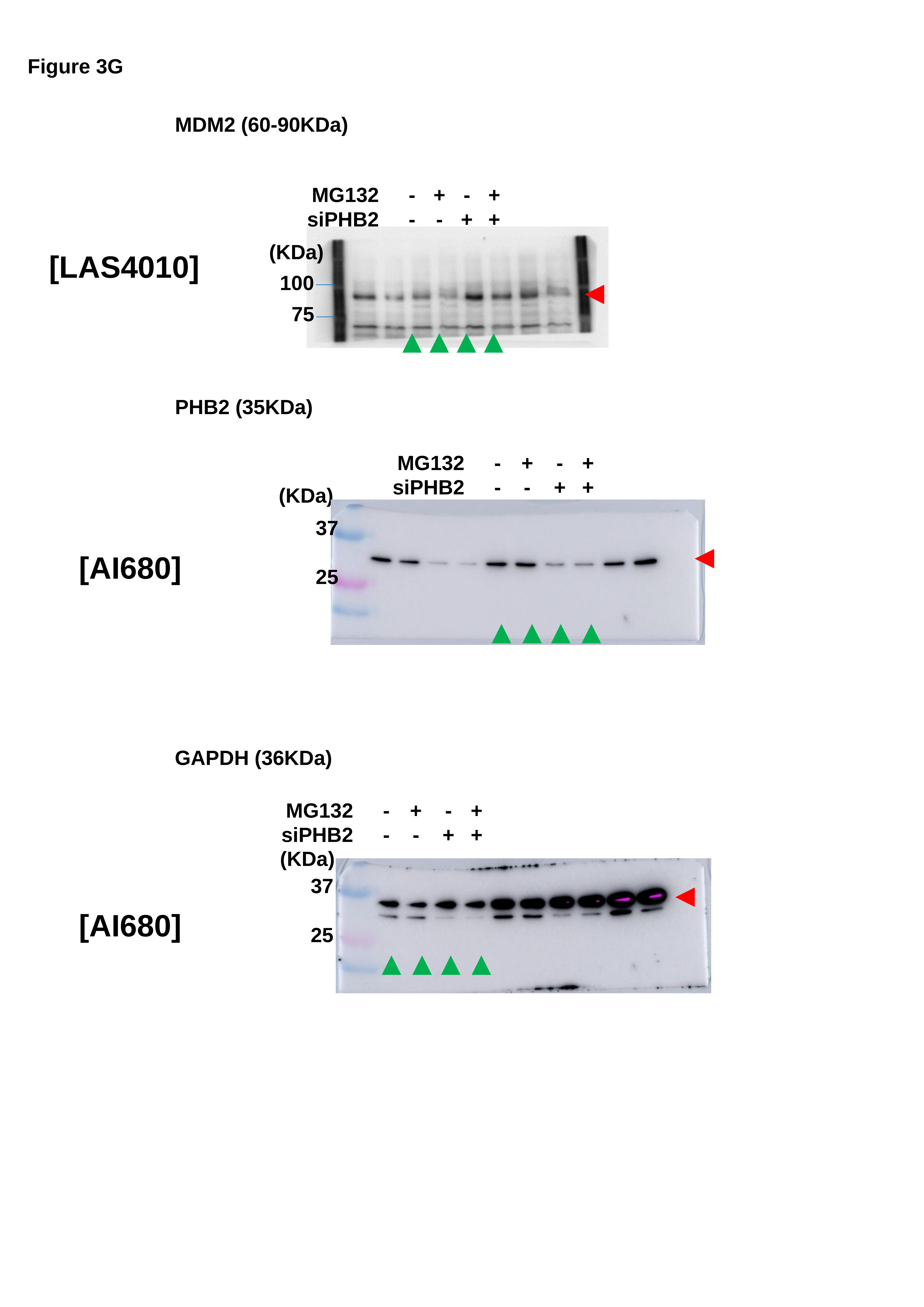

Figure 3G
MDM2 (60-90KDa)
MG132
-
+
-
+
siPHB2
-
-
+
+
(KDa)
100
75
[LAS4010]
PHB2 (35KDa)
MG132
-
+
-
+
siPHB2
-
-
+
+
(KDa)
37
25
[AI680]
GAPDH (36KDa)
MG132
-
+
-
+
siPHB2
-
-
+
+
(KDa)
37
25
[AI680]
