# Supplementary material for: Folate receptor α increases chemotherapy resistance through stabilizing MDM2 in cooperation with PHB2 that is overcome by MORAb‐202 in gastric cancer
Source: Clin Transl Med. 2021 Jun 1;11(6):e454. doi: 10.1002/ctm2.454 (PMC8167866; doi:10.1002/ctm2.454)
Supplement: Supplementary file 4 — Supplementary Table S1. Summary of FOLRα positivity in different histological subtypes of gastric cancer. [file CTM2-11-e454-s003.docx]

**Supplementary Table S1. FOLRα expression in gastric cancer of various histological subtypes**

| **Histology** | | **High positive** | **Low positive** | **Negative** |  |
| --- | --- | --- | --- | --- | --- |
| Intestinal | |  |  |  |  |
|  | Papillary adenocarcinoma | 1 | 0 | 0 |  |
|  | Tubular adenocarcinoma | 1 | 1 | 2 |  |
|  | Mucinous adenocarcinoma | 3 | 1 | 5 |  |
|  | Intestinal type | 2 | 0 | 3 |  |
| Diffuse |  |  |  |  |  |
|  | Signet ring cell adenocarcinoma | 1 | 4 | 31 |  |
|  | Infiltrating signet ring cell, mixed | 0 | 0 | 2 |  |
|  | Diffuse type | 0 | 0 | 3 |  |
| Mixed |  |  |  |  |  |
|  | Mixed tubular/papillary adenocarcinoma | 1 | 2 | 4 |  |
|  | Mixed mucinous/signet ring cell adenocarcinoma | 1 | 0 | 8 |  |
|  | Mixed hepatoid/signet ring cell adenocarcinoma | 0 | 0 | 4 |  |
|  | Mixed tubular/signet ring cell adenocarcinoma | 1 | 0 | 2 |  |
|  | Mixed mucinous/tubular adenocarcinoma | 0 | 0 | 2 |  |
|  | Mixed intestinal/diffuse type | 0 | 0 | 1 |  |
| Other |  |  |  |  |  |
|  | Hepatoid adenocarcinoma | 0 | 0 | 4 |  |
|  | Yolk sac–like adenocarcinoma | 0 | 1 | 0 |  |
|  | GIST | 0 | 1 | 1 |  |
|  | Subtype not defined | 7 | 1 | 32 |  |
| Total |  | 18 | 11 | 104 | 133 |
